# Evolutionary Dynamics of Human Papillomavirus in Thailand: Immunoinformatic Design of a Synergistic L1/L2 Vaccine Candidate

**DOI:** 10.3390/ph19050758

**Published:** 2026-05-12

**Authors:** Pornpiroon Nooroong, Rassanee Bissanum, Raphatphorn Navakanitworakul

**Affiliations:** Department of Biomedical Sciences and Biomedical Engineering, Faculty of Medicine, Prince of Songkla University, Hat Yai 90110, Thailand; pornpiroon.noo@gmail.com (P.N.); rassanee.b@gmail.com (R.B.)

**Keywords:** human papillomavirus (HPV), vaccine, IEDB database, immunoinformatics, cervical cancer

## Abstract

**Background/Objectives:** Current prophylactic human papillomavirus (HPV) vaccines rely on major capsid proteins (L1). Although highly immunogenic, L1-based immunity is clade-restricted owing to the hypervariability of HPV strains across different geographic regions. This restricts cross-protection against diverse, localized, and non-vaccine viral strains circulating in specific areas, leaving these populations vulnerable to regional genotypes. **Methods:** We aimed to design a cross-protective vaccine tailored to local viral diversity through evolutionary and immunoinformatic analyses of circulating HPV strains in Thailand. Both major (L1) and minor (L2) capsid proteins were analyzed to evaluate selective evolutionary pressures, structural sequence conservation, and cross-strain promiscuity of predicted B- and T-cell epitopes using the IEDB database. The predicted immunogenic profile of the combined L1/L2 proteins was further assessed using in silico immune response simulations. **Results:** Evolutionary analysis confirmed that although the L1 protein is under strict purifying selection, it exhibits high regional interclade variability. L1-only epitopes demonstrated restricted cross-strain conservation, creating structural blind spots against divergent regional variants. The minor capsid protein (L2) harbored highly conserved dual-action core peptides that are evolutionarily conserved across multiple HPV type. Incorporation of conserved L2 regions alongside L1 sequences may broaden the predicted epitope coverage and help address limitations associated with L1 variability. The proposed multi-targeted L1/L2 construct therefore represents a computational framework for the development of candidate cross-reactive HPV vaccines relevant to circulating Thai genotypes. However, experimental validation remains necessary to determine immunogenicity, cross-neutralization potential, and protective efficacy. **Conclusion:** Our findings highlight these conserved HPV epitopes as potential targets for future development of next-generation cross-subtype prophylactic vaccines with broader population coverage.

## 1. Introduction

Human papillomavirus (HPV) is the primary causative agent of cervical cancer and is associated with several other malignancies, including vulvar, vaginal, penile, anal, and head and neck cancers [1]. HPV is a double-stranded DNA (dsDNA) virus, and members of the genus Alphapapillomavirus are predominantly responsible for infections in humans. Both high-risk (HR) and low-risk (LR) HPV types are frequently reported in Thailand, and certain HR types contribute substantially to cervical, head, and neck cancers in both men and women [2,3,4,5,6,7,8,9]. Advances in next-generation sequencing have accelerated whole-genome characterization of multiple HPV types, including HPV 11, 16, 18, 31, and 35 [3,5,9,10]. The HPV genome is organized into an early (E) region encoding nonstructural proteins involved in replication, transcription, and oncogenesis (E1, E2, E4, E5, E6, E7, and E8) and a late (L) region encoding the structural capsid proteins L1 and L2 [11,12]. Despite this marked genomic and amino acid diversity, no novel HPV species have been reported in Thailand, reflecting the slow evolutionary rate of these viruses and their long coevolution with humans [13].

Current prophylactic vaccines are widely recognized as effective tools for preventing HPV infection and rely on L1-based virus-like particles (VLPs). Licensed vaccines, such as Cervarix, Gardasil-4, and Gardasil-9, induce high-titer type-specific neutralizing antibodies that confer strong protective immunity against primary infection [14,15,16]. However, their protection is largely limited to the included genotypes, and they lack therapeutic benefits for existing infections because L1 expression occurs late in the viral life cycle, allowing infected basal keratinocytes to evade cytotoxic T-lymphocyte responses [15]. Epidemiological analyses conducted in Thailand have suggested that targeting HPV 16 and 18 in combination with additional frequently detected HR types (HPV 45, 52, and 58) and LR types (HPV 6 and 11) could increase the predicted prevention rate of cervical cancer from approximately 74.5% to 87.6% [17]. Current bivalent vaccines are highly effective against HPV types 16 and 18; however, they exhibit limited cross-neutralization against other prevalent high- and low-risk genotypes, with only 32.8% coverage in the region. This underscores the need to incorporate conserved epitopes that can broaden the vaccine’s protective coverage [18].

A critical determinant of the success of next-generation vaccines is the efficient presentation of viral epitopes across diverse human leukocyte antigen (HLA) alleles. Failure to account for regional HLA polymorphisms can lead to severe immunological blind spots, drastically reducing population-level coverage and weakening the T-cell response. To overcome the structural constraints of L1-only formulations, vaccines must incorporate highly conserved viral domains that are capable of promiscuous HLA binding. The minor capsid protein (L2) represents a highly rational target; although naturally subdominant, its N-terminus contains evolutionarily constrained cross-reactive epitopes capable of inducing broad cellular and humoral responses across multiple HPV types.

Therefore, in this study, we aimed to (i) characterize the genetic and evolutionary diversity of HPV strains circulating in Thailand, (ii) evaluate structural sequence conservation and selective evolutionary pressures acting on all coding proteins, and (iii) computationally design a combined L1/L2 formulation. The primary novelty of our study lies in this region-specific framework. By integrating localized evolutionary dynamics with the highly prevalent HLA allele distribution unique to the Thai population, we aimed to design a tailored and complementary vaccine candidate. The developed formulation is enriched with highly promiscuous B- and T-cell epitopes, which are required to rescue regional HLA vulnerabilities, and it provides a rational strategy for developing a broadly cross-protective HPV vaccine tailored to the immunogenetic landscape of Southeast Asia, particularly Thailand.

## 2. Results

### 2.1. Retrieval of HPV Sequences and Evolutionary Analysis

**Figure 1:**
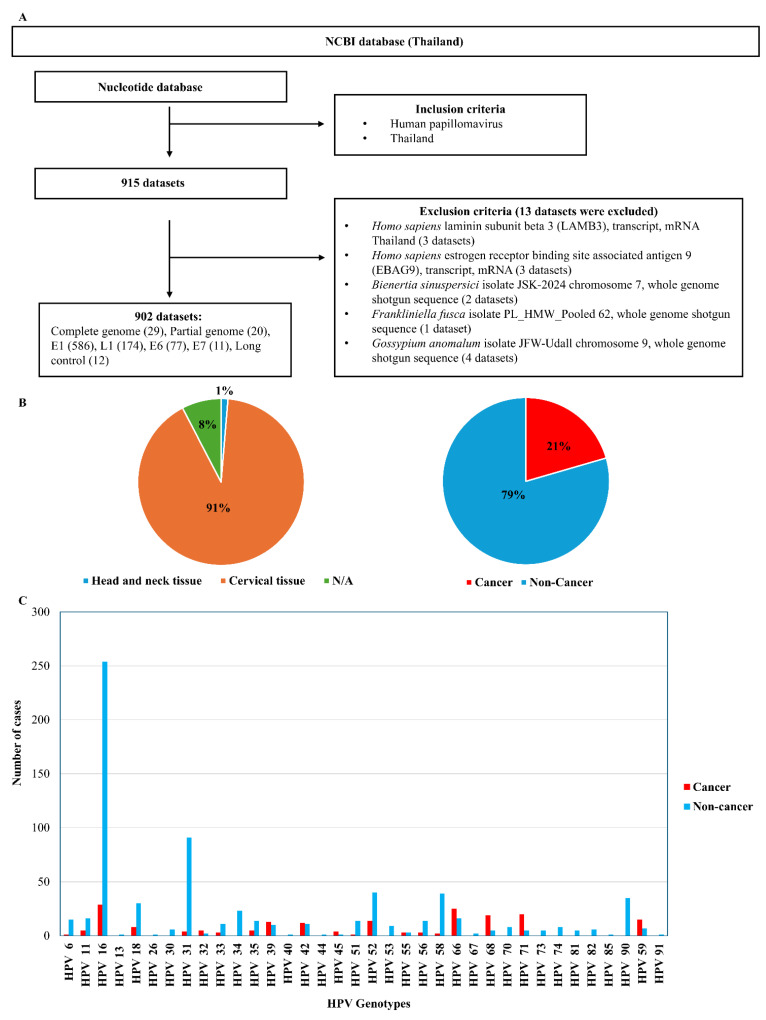
HPV data sources and genotype distribution. (**A**) Study workflow. (**B**) Data collection from publicly available databases across multiple anatomical sites. (**C**) Number of reported cases for each HPV genotype in cancer and non-cancer datasets. Data are derived from curated sources and are not intended for direct comparison between tissue types.

**Figure 2:**
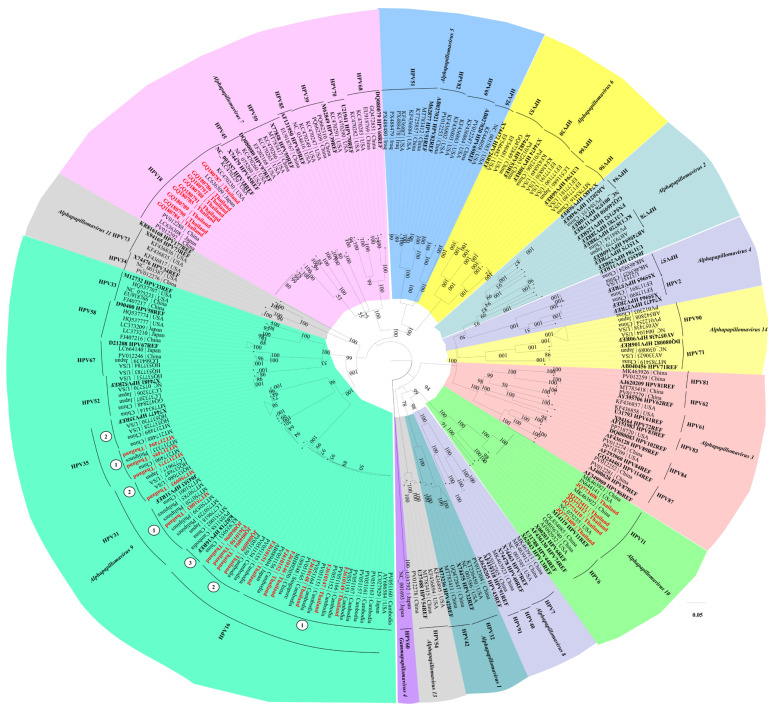
Phylogenetic analysis of the evolutionary relationships of Thai HPV genomes relative to reference strains from the PaVE database. Bold black indicates reference strains of PaVE database, and red text highlights the whole-genome HPV sequences identified in Thailand. However, HPV 16, 31, and 35 were separated from their reference HPV species as ①, ②, ③, reflecting evolutionary divergence within the HPV population in Thailand.

All HPV DNA and amino acid sequences recorded in Thailand were retrieved from the NCBI for Biotechnology Information database, whereas reference HPV sequences were obtained from the PaVE database. The corresponding accession numbers are listed in Table S1. The reported Thai HPV cases were classified according to clinical records and cross-checked with cervical cancer diagnoses (Figure 1). Phylogenetic reconstruction demonstrated that almost all Thai strains were clustered within the same clade as their respective reference HPV species from PaVE. However, HPV 16, 31, and 35 were separated from their reference HPV species, reflecting evolutionary divergence within the HPV population in Thailand (Figure 2).

### 2.2. Comparative Genomic Diversity and Evolutionary Constraints Across HPV Genes

**Figure 3:**
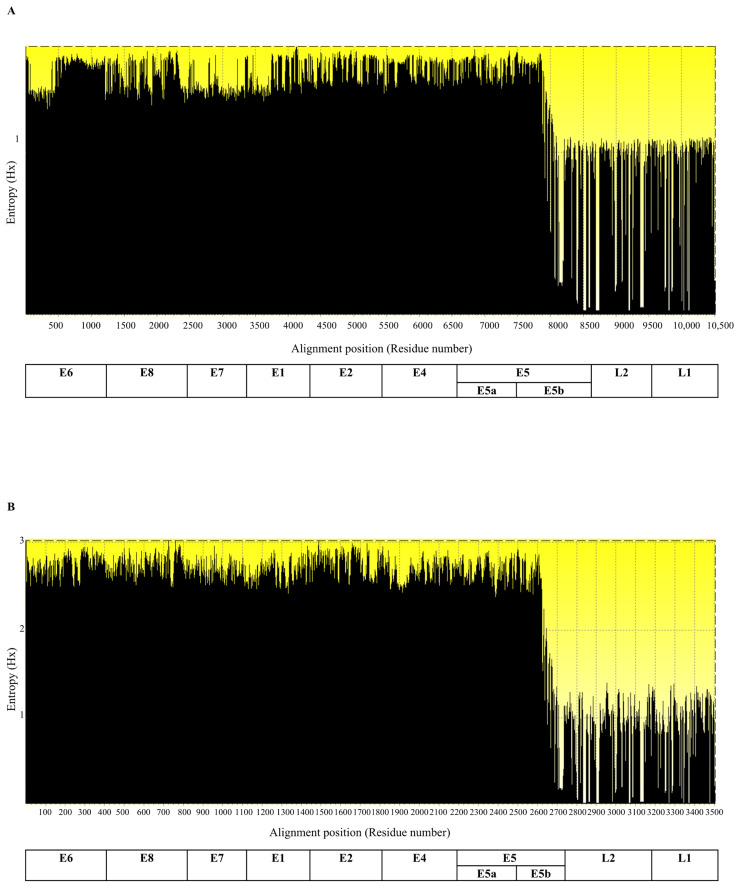
A quantitative assessment of the genetic variability (entropy) across the aligned sequences of multiple HPV strains recorded in Thailand, comparing both the nucleotide level (**A**) and the amino acid level (**B**).

To further assess variability among HPV strains, entropy profiling of both codon-based nucleotide and amino acid sequences consistently revealed lower genetic variation in L1 and L2 compared with that in other proteins (Figure 3A,B). For evolutionary analysis, whole-genome sequencing (WGS) and sequencing of each coding HPV gene were performed: WGS revealed an exceptionally high level of genetic diversity within the viral population, with high nucleotide diversity and haplotypes, alongside an average of 132.55 nucleotide differences (k) per sequence (Table 1). The Ka/Ks ratio < 1 signifies mild purifying (negative) selection to maintain essential viral functions. In addition, demographic neutrality tests provided a negative value for Fu’s F statistic (−18.283) and significantly positive values for Fu and Li’s D* (3.247, *p* < 0.02) and F* (2.349, *p* < 0.02) (Table 1). At the population level, this structural constraint was visualized in the L1 TCS haplotype network, which displayed a genetic divergence of HPV 6, 16, and 31 in the Thai population (light green-labeled node) (Figure S1), whereas the others were predominantly localized within the major ancestral central node (red labeled node). This indicates that some Thai L1 variants have undergone microevolution within the population while maintaining the amino acid composition of the L1 structural protein and influencing some differences in the amino acid composition profile (Figure S2).

Conversely, L2 and several genes expressed early (E1, E6, and E5), undergoing positive selection and rapid adaptation from a Ka/Ks ratio > 1 and a negative value for Fu’s Fs and Tajima’s D. Although specific early genes (E6, E7, and E2) had significantly positive Fu and Li’s D* and F* values, indicating balanced selection to maintain heterozygous variants in the population, the values of others remained neutral even though they displayed evolutionary variability (Table 1). In the haplotype network analysis, L2, E6, and E7 exhibited a highly complex topology connected by remarkably long branches with numerous mutational steps, and Thai sequences (cyan) were widely scattered across these distant clades and variable amino acid profiles (Figures S3 and S4), except the L2 Thai variants, which maintained the sequence shared with reference HPVs (Figures S5–S7). Moreover, L2 exhibited an evolutionary paradox of genomic variability at the proteomic level; it showed extreme sequence hypermutation of non-synonymous base substitutions and long mutational branches in the haplotype network, but the overall amino acid composition profile of L2 remained remarkably consistent. Its essential enrichment was consistently maintained in leucine (Leu), isoleucine (Ile), threonine (Thr), and proline (Pro) (Figure S8). The haplotype network for E1, E2, and E4 presented was continuous, messy, and scattered, especially that of E1 Thai variants, which were commonly separated from reference HPVs (Figures S9–S11), whereas the E5 haplotype network represented an intermediate evolutionary state with moderate fragmentation because of several subunits of E5 that were carried by each HPV (Figure S12). Consistently, the amino acid composition of the early genes significantly varied in each HPV strain (Figures S4, S13–S16).

L1 consistently displayed low nucleotide diversity, a strictly conserved (purifying selection) and stable structure, which maintained protein function. However, some gradual changes in some amino acid profiles occurred owing to microevolution, especially in some Thai variants, which can affect specific vaccine immune efficacy against newly emerging or unrepresented viral variants in the future. In contrast to both L1 and the early genes, the minor capsid protein L2 presents a unique evolutionary paradox that makes it a highly attractive candidate for a broadly cross-protective vaccine with an extreme genomic hypermutation of non-synonymous base substitutions designed to evade host immune surveillance, despite the overall amino acid composition profile of L2 remaining remarkably stable and conserved.

### 2.3. B- and T-Cell Epitope Prediction and Selection

Based on the high conservation of the L1 and L2 protein sequences, nine HPV protein vaccine candidates were selected using UniProt data, physicochemical suitability, and predicted solubility (Table S3). The most prevalent HPV type in Thailand and commercially used L1-based vaccines were also considered in the selection. Putative B-cell epitopes were identified using multiple complementary algorithms, including the BepiPred linear epitope, Chou and Fasman beta-turn, Emini surface accessibility, Karplus and Schulz flexibility, Kolaskar and Tongaonkar antigenicity, Parker hydrophilicity, and continuous B-cell epitopes (ElliPro). Several predicted epitopes that overlapped with conserved regions and showed antigenicity, no allergy, and no autoimmunity were selected as whole protein candidates with potential cross-reactivity with HPV types reported in Thailand. In conclusion, L2-based proteins were found in the cross-reaction of all HPV strains in Thailand, including HPV40, which is still present in Thailand and China (Tables S4 and S5).

For T cell immunity, MHC Class I cytotoxic T lymphocyte (CTL) and MHC Class II helper T lymphocyte (HTL) epitopes were predicted using the same proteins. Computational prediction of T cell epitopes confirmed that both major (L1) and minor (L2) capsid proteins are highly immunogenic. Across all the analyzed high- and low-risk HPV genotypes, both L1 and L2 yielded a robust range of high-affinity binding peptides for MHC class I and II molecules (MHC IC50 < 50 nM) (Tables S6–S9). Although L1 had a significantly higher number of predicted epitopes than L2, L2 had the most conserved region between MHC Class I and II compared to L1 (Tables S10 and S11). Notably, our combined immunogenicity and conservation analyses revealed that the L2 protein provides an important basis for broad cross-strain protection compared with L1. L2 yielded highly conserved dual-action core peptides capable of simultaneously inducing both MHC class I and II immune responses in multiple divergent strains. Specifically, regarding the L2 protein, the low-risk types (HPV 6 and 11) harbored the shared core peptides GSGFYLHPAW and YFARKRRKR across both MHC presentation pathways. Similarly, multiple high-risk HPV types (HPV 16, 31, 33, and 58) shared the highly conserved L2 core peptides YMLRKRRKRL and FVLHPSYFI. In contrast, cross-reactivity in the L1 protein was severely restricted; L1 yielded only two cross-strain dual-action cores, YYHAGTSRL and RKFLLQAGYRA, which were strictly confined to specific clades (HPV 16, 18, and 45 and HPV 31, 33, and 52, respectively).

To further maximize vaccine efficacy and prevent unnecessary sequence elongation, we systematically mapped the predicted linear B-cell epitopes against MHC-restricted T-cell epitopes to identify overlapping “immunological hotspots.” These regions are highly advantageous, as they act as dual-functioning core peptides capable of simultaneously priming both humoral (antibody) and cellular (T-cell) immune pathways. Several strain-specific B cell/T cell overlaps in the major capsid protein (L1) have been identified. For instance, in HPV 16, the B-cell epitope TVYLPPVPVSKVVSTDEY completely encompassed the MHC I epitope YLPPVPVSKV, whereas the B-cell epitope YIKMVSEPYG overlapped with the MHC II targets YPDYIKMVSEPYGDS and KYPDYIKMVSEPYGD. Similar dual-action MHC I overlaps were identified across other cables, including HPV 11 (FALPDSSLF), HPV 18 (FGLPDTSIY), and HPV 45 (SPSPSGSII). Remarkably, despite being naturally subdominant and having a low molecular weight, the minor capsid protein (L2) exhibited a densely concentrated array of dual-action potential regions. In HPV 6, extensive B-cell epitopes seamlessly overlap with multiple MHC I targets (e.g., GPMARPPVV and TPNTVTQPW) as well as the MHC II target GHILISAPTVTSHPI. Comparable high-density B/T cell overlapping hotspots were distinctly observed in HPV 11 (e.g., FPTASMGTPF), HPV 16 (VPSVPSTSL), and HPV 31 (ASATTTSTL). This widespread overlap mathematically reinforces the value of L2 in rational vaccine design; it harbors dense, highly efficient immunological targets capable of driving robust cross-pathway immunity within a highly compact sequence.

### 2.4. Population Coverage of Predicted MHC Class I and II Epitopes

To evaluate the global and regional efficacies of the predicted epitopes, HLA population coverage analysis was conducted. For MHC class I, the L1-alone epitopes demonstrated exceptionally high coverage, theoretically protecting 99.93% of the global population and 99.89% of the Thai population. However, the combined L1/L2 multi-epitopes significantly enhanced the depth and redundancy of the immune response. Although the combined L1/L2 coverage increased slightly to 99.94% globally and to 99.90% in Thailand, the average hit rate, which represents the average number of distinct epitopes recognized by an individual’s HLA alleles, drastically increased. In the Thai population, the average number of hits increased from 36.79 (L1 alone) to 53.49 (L1/L2 in combination). This indicates that incorporating L2 facilitates a highly redundant, multi-target cytotoxic defense, reducing the likelihood of viral immune escape due to a single-epitope mutation (Table 2).

The most significant advantage of the combined L1/L2 formulation was observed in MHC Class II presentation; L1-alone epitopes exhibited moderate Class II coverage, with 54.64% of the global population and 44.94% of the Thai population. By integrating the highly conserved, structurally constrained dual-action epitopes of L2, the global Class II population coverage increased to 70.55%. Regionally, this synergistic effect was particularly noteworthy. In Thailand, the combined L1/L2 construct increased Class II coverage by 55.89%, with the average number of presented epitopes rising from 5.27 to 7.98 per individual. Similar improvements were observed across all major geographic regions, including East Asia (62.55% to 76.32%) and North America (58.16% to 76.66%) (Table 2).

### 2.5. Polymorphism of HLA Alleles

**Figure 4:**
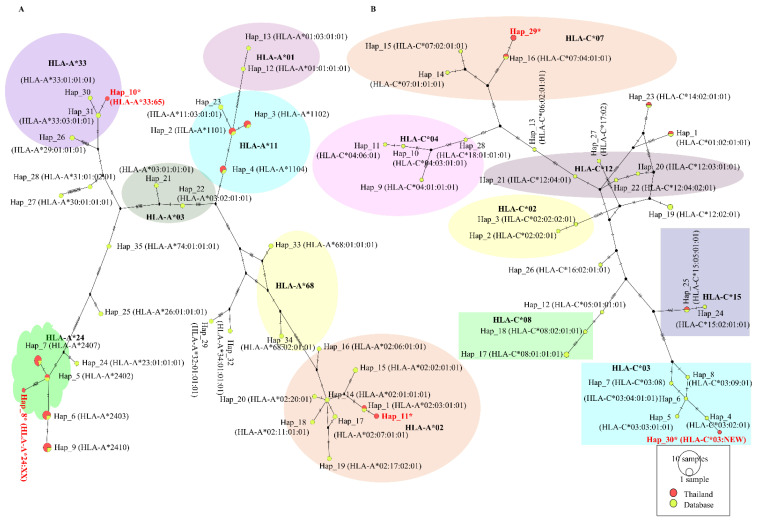
Distribution of human leukocyte antigen A (HLA-A) (**A**) and HLA-C (**B**) allele frequencies in a localized Thai cohort compared to references from databases. Bold black indicates reference HLA, and red text highlights the most divergent haplotypes in Thailand.

Since the IEDB database has historically been analyzed for Caucasian/European HLA frequencies, relying solely on its default outputs can lead to misleading results or bias regarding rare or underrepresented Thai HLA alleles. An analysis of the localized sequencing data revealed a distinct MHC Class I profile within the Thai population, predominantly characterized by high frequencies of specific HLA-A haplotypes, notably HLA-A*11 (e.g., A*11:01, A*11:02, A*11:03), HLA-A*24 (e.g., A*24:02), HLA-A*02 (e.g., A*02:03, A*02:06), and HLA-A*33, but the HLA Thai variant harbored some different and distant HLA alleles, including HLA-A*02, A*24:XX, A*33:65, which are not included in common HLA frequency data in the IEDB database (Figure 4A). However, cross-referencing these regional alleles against our immunoinformatics predictions demonstrated that both the L1 and L2 proteins harbored strong-binding epitopes as well as highly conserved targets (such as the YMLRKRRKRL core) of the L2 protein candidate that can bind more to HLA-A*02:01 and HLA-A*33:01 alleles, increasing the average number of presented epitopes per Thai individual from 36.79 to 53.49. In contrast to HLA-B, which showed no polymorphisms among Thai variants compared to the common HLA-B, HLA-C haplotypes showed divergence from HLA-C variants 03 and 07 (Figure 4B). Cross-referencing of these specific regional haplotypes using our immunoinformatics data revealed a fascinating divergence in the binding efficiency between the major and minor capsid proteins. Moreover, the highly immunogenic L1 protein elicited numerous distinct HLA-C-binding interactions (76 peptide allele hits across 20 HLA-C variants). In contrast, the highly constrained L2 protein yielded far fewer distinct HLA-C targets (13 distinct hits). However, computational analysis demonstrated that these specific and highly conserved L2 targets were bound to the same 20 distinct Thai HLA-C alleles covered by L1 (Tables S10 and S11).

**Figure 5:**
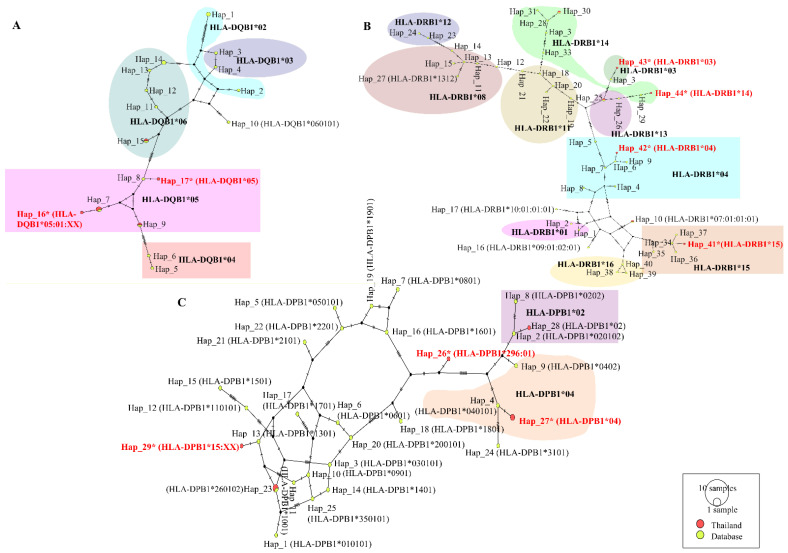
Distribution of human leukocyte antigen DQ (**A**), DR (**B**), and DP (**C**) (HLA- DQ, DR, and DP) allele frequencies in a localized Thai cohort compared to references from databases. Bold black indicates reference HLA, and red text highlights the most divergent haplotypes in Thailand.

The distinct divergence between the L1 and L2 predictive profiles became evident when evaluating the MHC Class II presentations against the localized Thai haplotypes. Regional sequencing identified specific endemic Class II alleles that were the most divergent within the HLA-DQB1 (DQB1*05), HLA-DRB1 (DRB1*03, DRB1*04, DRB1*14, and DRB1*15), and HLA-DPB1 (DPB1*04, DPB1*15, and DPB1*296:01) loci (Figure 5A–C). Our data demonstrated that an L1-only vaccine formulation would be structurally inadequate to cover this specific regional Class II diversity. The high clade restriction of L1 epitopes resulted in inconsistent binding to the Thai DQB1 and DPB1 variants, resulting in severely restricted regional Class II coverage of only 44.94% (compared to 54.64% global coverage). The resolution of this demographic blind spot resides unequivocally within the minor capsid protein (L2). The identified L2 dual-action cores, particularly the highly conserved LHPSY motif, demonstrated exceptional conservation and successfully established binding predictions with regionally critical alleles, such as HLA-DQA1*01:01/DQB1*05:01, across multiple viral genotypes (e.g., HPV 16, 52, and 58) (Table S11). By effectively bridging the binding gap for these Thai-specific Class II haplotypes, the integration of L2 synergistically rescued localized population coverage, increasing it to 55.89%, and increasing the average epitope hits from 5.27 to 7.98 (Table 2).

### 2.6. Immune Response Simulation

**Figure 6:**
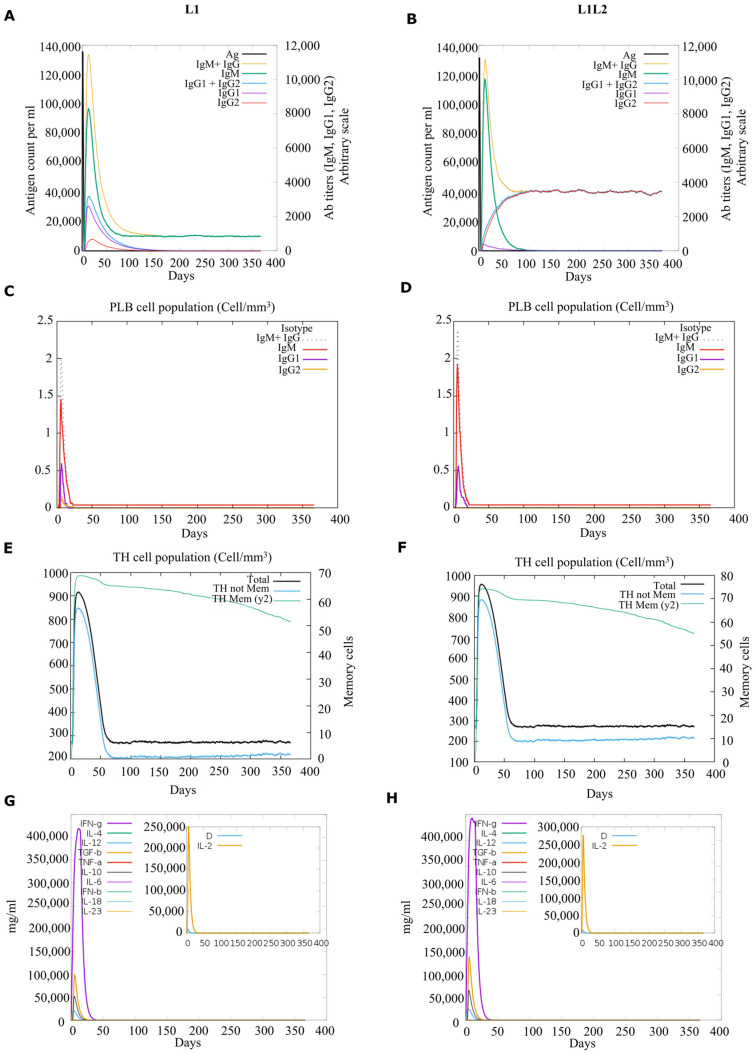
Computational models comparing the immunogenic profiles of the L1-only protein (**A**,**C**,**E**,**G**) against the combined L1/L2 capsid protein (**B**,**D**,**F**,**H**). (**A**,**B**) Simulated humoral immune responses demonstrating profound antigen-induced antibody production, characterized by initial IgM peaks and subsequent robust class switching to high-affinity IgG (IgG1 and IgG2) isotypes. (**C**,**D**) B-lymphocyte population dynamics, illustrating active cellular expansion and the successful generation of durable memory B-cell pools. (**E**,**F**) CD4+ Helper T-Lymphocyte (TH) population dynamics, confirming sustained activation and immunological memory generation. (**G**,**H**) Illustration of the concentration kinetics of cytokines that regulate the immune response balance (e.g., IFN-γ, IL-2, IL-6, IL-10, and TGF-β).

To computationally validate and compare the immunogenic profiles of L1 alone and combined L1 and L2 capsid proteins, simulations were configured utilizing the specific, highly prevalent HLA alleles identified in the analysis of MHC class I and II (e.g., A*02:01, A*33:01, A*68:01, B*44:03, B*53:01, DRB1*01:01, and DRB3*01:01). The integration of the L1 and L2 capsid proteins did not cause antigenic competition or dilute the humoral response; instead, it generated an equally massive sustained profile of high-affinity IgM and IgG (specifically IgG1 and IgG2) essential for viral neutralization, compared to L1 alone (Figure 6A,B). The B cell population dynamics confirmed that the combined L1/L2 capsid proteins (Figure 6D) successfully promoted active B cell expansion and established a durable memory B cell pool that exceeded the highly immunogenic L1 baseline (Figure 6C). Critical validation of the combined L1/L2 capsid proteins was observed in the MHC Class II pathway, which sustained helper T cell activation more than L1 alone (Figure 6E,F). Both the L1 and combined L1/L2 capsid proteins successfully induced a robust pro-inflammatory microenvironment, which is essential for driving cellular (Th1) immunity against intracellular viral pathogens; however, the combined L1/L2 capsid proteins promoted IL-2 secretion significantly more than L1 alone (Figure 6G,H).

## 3. Discussion

HPVs are primarily sexually transmitted pathogens [1]. In Thailand, HPV16 and HPV18 are the most prevalent high-risk types, responsible for approximately 67.6% of cervical cancer cases; additional common high-risk genotypes include HPV31, 39, 45, 58, and 66 [2,4,5,8,19,20,21]. In the general population, non-oncogenic HPV types, such as HPV90 (16.6%) and HPV71 (10.3%), are frequently detected [2,22]. Whole-genome sequences of HPV11, 16, 18, 31, and 35 from multiple anatomical sites (cervix, larynx, and lungs) have recently been reported in Thailand [3,7,9,10]. Furthermore, HPV infection is being increasingly recognized among Thai males with anogenital warts [23]. Despite vaccines being available, incomplete vaccination coverage and limited vaccine accessibility in some regions contribute to continued HPV transmission and HPV-associated cancers [18,24,25,26]. Based on our analysis (Figure 1), some high-risk HPV genotypes appear less frequent in cancer-associated datasets than in non-cancer datasets. This likely reflects database-related biases, including unequal sequence representation and sampling differences, rather than reduced oncogenic potential. Therefore, these findings should be interpreted with caution, as genotype frequency does not directly reflect carcinogenic risk.

Our phylogenetic reconstruction confirmed that the majority of Thai HPV strains clustered with established global clades (as defined by the PaVE reference database); however, high-risk genotypes, specifically HPV 16, 31, and 35, demonstrated distinct, localized separation. This clustering, separate from the standard reference genomes, suggests that these high-risk oncogenic strains underwent localized evolutionary adaptation driven by geographic selective pressures. This geographic divergence is consistent with the findings of previous global epidemiological reports that categorized high-risk HPV types 16 and 52 into distinct geographic lineages [20,21].

Comprehensive comparative analyses revealed that the structural proteins L1 and L2 exhibited amino acid conservation, although the viral genome did not evolve uniformly. These haplotype networks corroborated our statistical evolutionary metrics. The calculated Ka/Ks ratios and neutrality tests demonstrated a contrast between the structural genes (L1 and L2), which regulate viral entry, and the early genes (E6, E7, and E2), which drive intracellular oncogenesis and immune evasion. The strict purifying selection observed in L1 aligns with the findings of previous studies showing that any non-synonymous mutation disrupting capsid structural integrity is rapidly eliminated, resulting in an excess of rare, neutral variants indicated by negative Fu Fs values [22,23,24,25]. Topologically, this structural constraint is displayed in the L1 haplotype network as a classic “star-like” structure, in which most sequences group within a dominant ancestral node, with some divergent branches representing localized Thai microevolution reflected in amino acid composition similar to patterns observed in China and Greece [26,27]. In contrast, L2 exhibited positive selection, because its N-terminus is transiently exposed to host-neutralizing antibodies during endosomal entry and viral uncoating; therefore, it remains under intense selective pressure [28,29]. Our analysis identified significant balancing selection within the E6, E7, and E2 genes. Although the overall genome showed non-significant Fu and Li’s D* and F* values, E6 (D* = 2.05), E7 (D* = 2.77, F* = 1.81), and E2 (D* = 1.87) yielded significantly positive results, indicating the active maintenance of multiple distinct alleles within the population rather than a single advantageous mutation sweeping to fixation, whereas the others remained neutral even with high genetic variability [23,30]. This heterozygote advantage is a common viral adaptation to host HLA diversity, as observed in viruses such as the hepatitis C virus (HCV) [31]. The loss of heterozygosity in human HLA alleles has been directly linked to cervical cancer development [32,33].

A broadly efficacious next-generation HPV vaccine must establish multiple-HPV immunity that exceeds that of current prophylactic formulations. Current commercial vaccines (Gardasil-9 and Cervarix) rely entirely on L1 VLPs. Although these vaccines generate massive titers of neutralizing antibodies, our immunoinformatics analysis further demonstrated the primary biological limitation of L1: its highly immunogenic B-cell epitopes are located on hypervariable, surface-exposed loops that are strictly clade-restricted, offering minimal cross-protection against nonvaccine strains [34,35]. Using a multi-algorithmic approach, we exclusively identified L2-based candidate proteins that demonstrated broad theoretical cross-reactivity across all prevalent Thai HPV genotypes, including deeply divergent nonvaccine strains, such as HPV40. This aligns with findings from in vivo model studies demonstrating that conserved L2 neutralization epitopes (e.g., RG-1) can elicit cross-neutralizing antibodies across diverse papillomavirus genera [29,36].

Although B cell responses block initial viral entry, clearing established intracellular infections requires robust cellular immunity [37]. Effective viral clearance requires the simultaneous activation of CD8+ CTLs to destroy infected cells and CD4+ HTLs to sustain the response [38]. Our computational predictions confirm that L2 harbors highly conserved “dual-action” core peptides capable of engaging both pathways across vastly divergent strains. For instance, low-risk types (HPV 6 and 11) share the GSGFYLHPAW and YFARKRRKR cores, whereas major high-risk types (HPV 16, 31, 33, and 58) share the YMLRKRRKRL and FVLHPSYFI cores [29]. These L2 sequences are evolutionary constraints due to their mechanical roles in nuclear transport; however, genetic variability enables escape from host immune detection. Conversely, L1 yielded only two cross-strain dual-action cores that were strictly confined within specific clades (Alphapapillomavirus-7 and -9), rendering L1 structurally incapable of bridging distinct evolutionary branches [39]. This localized evolutionary tracking is particularly relevant given the high circulating prevalence of diverse low-risk HPV genotypes observed among high-risk demographics in Thailand, such as female sex workers, transgender individuals, and men who have sex with men. Currently, no accessible vaccines are available for this population, and existing vaccines do not provide coverage against diverse nonvaccine strains [40,41].

Our analysis of localized sequencing data revealed critical immunogenetic vulnerabilities in the Thai cohort. We identified a highly endemic MHC profile that was absent from global databases, including the divergent DQB1*05, DPB1*04, and DPB1*:296:01 lineages that are largely absent from global databases. This regional approach allowed us to overcome the inherent dataset limitations of public repositories, which are historically skewed toward Caucasian and European immunogenetic profiles. Ancestrally inherited host genetic profiles heavily influence viral clearance, and the HLA-B*07:02 and HLA-C*07:02 alleles inherited from archaic Neanderthal introgression are strongly associated with cervical cancer risk [42]. Our localized sequencing confirmed the absolute conservation of the HLA-B and C, particularly C *07:02, in Thailand [43]. The clinical impact of regional HLA polymorphisms is primarily driven by their peptide-binding affinities. Specific high-risk alleles, such as HLA-DRB1*13:02, HLA-DQB1*05:02, and HLA-DRB1*03:01, which were highly prevalent in our cohort, were associated with persistent hrHPV infections due to their significantly weak binding affinities for hrHPV-derived peptides [44]. Consequently, relying solely on L1 epitopes can create immunological blind spots. Our models showed that L1-only epitopes failed to consistently bind the Thai MHC Class II variants, resulting in inadequate regional Class II coverage of merely 44.94% (compared to 54.64% globally), leaving massive portions of the Southeast Asian demographic without sufficient CD4+ activation. This mirrors adjacent regional models, where L1 cores (e.g., LRRRPTIGP, which is similar to the L1 peptide core identified in this study) yielded severely limited coverage of 25.65% in Western Japanese populations [45]. Integration of the L2 minor capsid protein may resolve this demographic blind spot. The highly conserved L2 dual-action cores (specifically the LHPSY motif) exhibited exceptional universality, establishing strong binding predictions with these regionally critical binding alleles (e.g., HLA-DQA1*01:01/DQB1*05:01). This integration synergistically rescued localized population coverage, elevating Thai Class II coverage from 44.94% to 55.89% and increasing the average epitope hits from 5.27 to 7.98 per individual. Furthermore, a broadly effective vaccine must successfully engage with the natural protective genetic architecture of the host. Epidemiological data from genetically proximate Han Chinese populations highlight that specific alleles, such as HLA-C*03:02 and DPB1*05:01, confer robust natural resistance against disease progression across diverse HPV genotypes [46,47]. Although standard L1 formulations failed to bind these naturally protective targets owing to their structure, our analysis proved that the conserved L2 core peptides robustly engage both HLA-C*03:02 and DPB1*05:01. In addition, our analysis revealed a profound immunological advantage of the minor capsid protein L2. Historically, L2 has been considered sub-dominant in natural infections because it is largely shielded by L1 on the surface of mature virions. However, when isolated in silico, we found that L2 exhibited a remarkably high density of broadly conserved B- and T-cell overlapping regions relative to its small molecular weight. For instance, highly dense B/T cell overlaps have been identified across both low-risk (HPV 6 and 11) and high-risk (HPV 16 and 31) strains within the L2 sequence [48,49].

These structural advantages were further explored using in silico immune response simulations. The combined L1/L2 formulation did not cause antigenic competition or dilute the primary humoral response; rather, it effectively sustained IgM and IgG (both IgG1 and 2) and CD4+ TH populations and significantly elevated IL-2 secretion compared to an L1-only baseline. These computational findings are consistent with observations reported in recent in vivo studies of chimeric vaccine models [48,49,50,51,52,53] and suggest that a combined L1/L2 strategy may provide a rational framework for expanding predicted immune breadth in genetically diverse and resource-limited settings. However, experimental validation remains necessary to confirm these predicted immunological effects.

## 4. Materials and Methods

### 4.1. Data Resources

**Figure 7:**
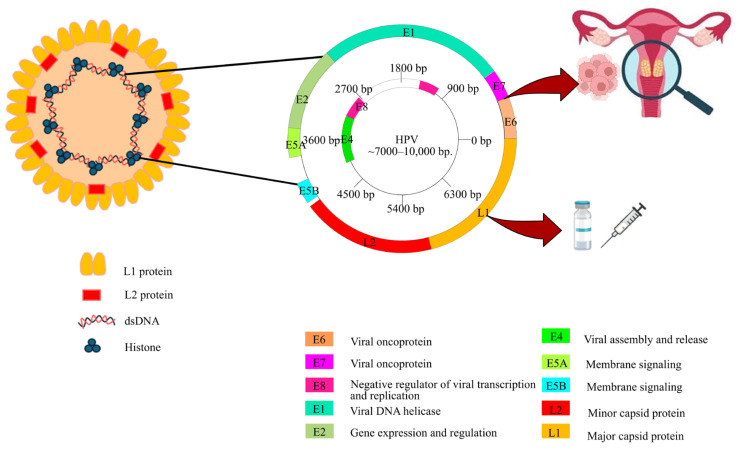
The structure of the human papillomavirus (HPV) virion, the organization of its circular double-stranded DNA (dsDNA) genome, and the roles of its various proteins in disease and vaccine development.

Publicly available reference HPV genomes (*n* = 177) were downloaded from the papillomavirus episteme database on 16 June 2025 [54]. Additional 902 HPV sequences recorded in Thailand were searched using the “Virus/Taxonomy” filter with specific taxonomic identifiers, as shown in Figure 1. All the filtered data were retrieved from the National Center for Biotechnology Information (NCBI) database [55]. Furthermore, other HPV sequences that aligned with our strains from various countries, including Vietnam, Cambodia, the Philippines, Singapore, Malaysia, Indonesia, China, Japan, Iraq, and the USA, were selected as candidates. The HPV genome was annotated according to its canonical organization, comprising an early (E) region encoding non-structural proteins involved in replication, transcription, and oncogenesis (E1, E2, E4, E5, E6, E7, and E8), and a late (L) region encoding the structural capsid proteins L1 and L2 (Figure 7).

### 4.2. Multiple Sequence Alignment and Evolution Analysis

Complete HPV genomes, including representative variant lineages/sublineages and all Thai-reported sequences, were aligned using MUSCLE with default parameters. Subsequently, each coding region was codon-aligned and compared with the amino acid alignment using MEGA 12 [56]. Genetic and amino acid variabilities were assessed using the entropy method in BioEdit v7.0.0 [57].

Phylogenetic relationships between the reference and Thai HPV strains were reconstructed using the maximum likelihood method under the GTR+G+I model with 1000 bootstrap replicates in MEGA 12 [56]. Additionally, each coding gene reported in Thailand was aligned and assessed for nucleotide diversity, nucleotide differences, number of variable sites, number of haplotypes (h), haplotype diversity (Hd), synonymous and nonsynonymous substitution rates, and neutrality using DnaSP v6.0 [58]. All coding gene haplotypes were constructed with a haplotype network to visualize the genetic variation connected by mutations using Templeton–Crandall–Sing (TCS) analysis in PopArt 1.7 [59].

### 4.3. B-Cell Epitope and MHC Class I-II Analysis

Linear B-cell epitopes were predicted using the IEDB BepiPred tool and evaluated alongside physicochemical parameters, including Chou–Fasman beta-turns, Emini surface accessibility, Karplus–Schulz flexibility, Kolaskar–Tongaonkar antigenicity, and Parker hydrophilicity (https://tools.iedb.org/bcell/; accessed on 16 June 2025) [60]. Antigenicity and allergenicity were also predicted using VaxiJen v2.0, with a threshold score of 0.4 and AllerTOP v2.1 (https://www.ddg-pharmfac.net/allertop_test/; accessed on 16 June 2025), respectively [61,62]. An autoimmune assessment was conducted by aligning (BLASTp) the candidate proteins against the NCBI *Homo sapiens* database to identify potential cross-reactivity. Subsequently, antigenic proteins were analyzed using ProtParam (https://web.expasy.org/protparam/; accessed on 16 June 2025) [63] to identify candidates with favorable physicochemical characteristics: molecular weight 40–110 kDa, thermostability (aliphatic index 66.5–84.3), stability, acceptable estimated half-life, and hydrophilicity (GRAVY < 0), to enhance solubility and biological performance. To identify conformational B-cell epitopes, the 3D structure of all candidate proteins was analyzed using the ElliPro server available at IEDB (https://tools.iedb.org/ellipro/; accessed on 16 June 2025) with a threshold score of 0.5 and a maximum residue distance of 6 Å.

Predicted CTL epitopes (9–10 mers) were assessed using NetMHCpan 4.1 EL across frequent MHC class I supertypes (A1, A2, A3, A11, A23, A24, A26, A30, A31, A32, A33, A68, B7, B8, B15, B35, B40, B44, B51, B53, B57, B58, C1, C2, C3, C4, C5, C6, C7, C8, C12, C14, C15, C16, and C17) [64]. The predictions included proteasomal cleavage, TAP transport efficiency, and peptide–MHC binding. Naturally processed peptides were assessed using MHC-NPs. Epitopes were retained when concordant across methods, exhibiting high TAP transport scores and strong predictive processing. Peptides with the lowest percentile ranks, a predicted half-maximal inhibitory concentration (MHC IC50) of <50 nM (indicating stronger binding affinity), and high cumulative prediction scores (prob and total scores) were considered the strongest candidate epitopes for eliciting a CD8+ CTL immune response [65].

HTL epitopes (15-mers) were predicted using NetMHCII 2.3 for HLA-DR, HLA-DQ, and HLA-DP alleles [66]. Binding affinities were classified as high (IC50 < 50 nM), intermediate (IC50 < 500 nM), or low (IC50 < 5000 nM). Epitopes were further evaluated using MHCII-NP and allele-independent CD4+ T-cell immunogenicity predictors. The highest-affinity epitopes and their corresponding alleles were selected for population coverage analysis.

### 4.4. Population Coverage Analysis

Given the geographic heterogeneity of HLA allele frequencies, the population coverage for selected CTL and HTL epitopes was estimated using the IEDB population coverage tool for MHC class I, class II, and combined responses, both globally and specifically in Thailand [67].

### 4.5. HLA Polymorphic Variability

To ensure the reliability of population coverage estimates for Thailand, an HLA dataset specific to the Thai population was retrieved from the NCBI database and compared with the standard IMGT/HLA database. The datasets used in this study were downloaded from the IPD-IMGT/HLA database (https://www.ebi.ac.uk/ipd/imgt/hla; accessed on 16 June 2025) using the accession numbers listed in Table S2. To compare the Thai HLA dataset with the official IMGT/HLA database, haplotype data were generated using polymorphism analysis performed in DnaSP v6.0 [58]. Subsequently, a haplotype network was constructed using TCS analysis in PopART v1.7 [59].

### 4.6. In Silico Immune Response Assessment

Immune responses to candidate constructs were simulated using CImmSim [68] (http://kraken.iac.rm.cnr.it/C-IMMSIM/, accessed on 16 June 2025). HLA settings reflected prevalent alleles identified in our dataset (A*02:01, A*33:01, A*6801, B*4403, B*53:01, B*57:01, DRB1*0101, DRB301:01, and DRB3*02:02). The simulator integrates AI-based modeling and position-specific scoring matrices to evaluate epitope processing and immune interactions [19]. The following parameters were used as defaults for the simulations: 1100 simulation steps, random seeds 12,345, vaccine without LPS, adjuvant value 100, and simulation volume 50. The magnitude of the immune response was quantified using C-ImmSim output metrics.

## 5. Conclusions and Limitations

Limited accessibility to prophylactic HPV vaccines in Thailand for certain populations, particularly sex workers and transgender individuals, contributes to the persistent circulation of diverse viral strains and an ongoing burden of associated cancers. Immunoinformatics provides a rapid and cost-effective strategy for identifying broadly protective vaccine candidates tailored to local viruses and host HLA diversity.

In this study, we found that although the major capsid protein L1 is highly immunogenic, its clade-restricted nature may lead to incomplete immunological coverage in Thai individuals. By incorporating conserved and potentially promiscuous B- and T-cell epitopes from the L2 minor capsid protein, a combined L1/L2 formulation may improve predicted population coverage and help address HLA-associated binding limitations. This shift from a conventional L1-focused prophylaxis to a combined L1/L2 strategy represents a rational approach to enhance cross-protective potential in Southeast Asia, although experimental validation is required to confirm these findings.

However, several important limitations should be acknowledged. All findings are derived from computational predictions and in silico immune simulations, which cannot fully capture the complexity of antigen processing, immune regulation, or vaccine-induced protection in vivo. In addition, key factors such as structural stability, protein expression, manufacturability, and safety have not been experimentally evaluated. Therefore, the proposed vaccine construct should be considered hypothesis-generating. Future studies are required to validate these findings through experimental approaches, including epitope-specific HLA-binding assays, in vitro immunogenicity testing, structural characterization, and preclinical in vivo evaluation to determine whether the predicted advantages translate into measurable protective efficacy.

## Figures and Tables

**Table 1:** Genetic diversity analysis based on L1, L2, E1, E6, E4, E7, E5, and E2 genes.

Gene	Number of Samples (N)	Non-Synonymous Substitution Rates (Ka)	Synonymous Substitution Rates (Ks)	Ka/Ks	Nucleotide Diversity Per Site (Pi)	Average Number of Nucleotide Differences (k)	Number of Variable Sites (S)	Number of Haplotypes (h)	Haplotype Diversity (Hd)	Variance of Haplotype Diversity	Standard Deviation of Haplotype Diversity	Fu’s Fs Statistic	Tajima’s D	Fu and Li’s D* Test Statistic	Fu and Li’s F* Test Statistic
WGS	260	1.281	1.766	0.725	0.603	132.551	220	132.551	0.993	0.000	0.002	−18.283	0.8233	3.24764 **	2.34903 **
*L1*	1034	0.087	0.724	0.121	0.233	7.675	33.000	139.000	0.953	0.000	0.003	−106.599	−0.478	−1.084	−0.942
*L2*	243	0.507	0.120	4.218	0.270	26.146	21.000	69.000	0.840	0.000	0.021	−43.329	−0.525	1.156	0.493
*E1*	707	0.542	0.373	1.453	0.273	6.836	24.000	86.000	0.927	0.000	0.006	−32.083	−0.065	−0.341	−0.255
*E6*	841	0.491	0.388	1.266	0.315	29.273	86.000	98.000	0.780	0.000	0.012	0.416	−0.166	2.05365 **	0.943
*E4*	218	0.219	0.680	0.322	0.274	4.379	13.000	56.000	0.956	0.000	0.005	−40.434	0.010	0.167	0.124
*E7*	370	0.460	0.956	0.481	0.352	13.709	33.000	92.000	0.897	0.000	0.013	−25.518	0.173	2.77825 **	1.81129 **
*E5*	214	0.324	0.199	1.628	0.269	1.882	6.000	41.000	0.914	0.000	0.009	−33.336	−0.502	0.236	−0.052
*E2*	244	0.318	0.962	0.331	0.283	13.295	41.000	75.000	0.939	0.000	0.009	−21.528	−0.047	1.87437 **	1.169

Statistical significance: **, *p* < 0.02; Statistical significance: Not significant, *p* > 0.10.

**Table 2:** Global and regional HLA population coverage analysis for predicted L1 and combined L1/L2 multi-epitopes.

L1L2						
Population/Area	Class I			Class II		
	Coverage ^a^	Average Hit ^b^	pc90 ^c^	Coverage ^a^	Average Hit ^b^	pc90 ^c^
Thailand	99.90%	53.49	31.35	55.89%	7.98	0.23
World	99.94%	56.01	34.09	70.55%	10.15	0.34
East Asia	99.97%	60.92	37.99	76.32%	9.11	0.42
Northeast Asia	99.63%	50.62	28.52	51.69%	6.48	0.21
South Asia	98.91%	43.4	19.87	64.18%	10.66	0.28
Southeast Asia	99.90%	53.25	31.1	48.55%	5.61	0.19
Southwest Asia	98.30%	39.48	15.87	35.64%	4.72	0.16
Europe	100.00%	61.53	42.98	73.82%	11.43	0.38
North America	99.96%	57.88	35.24	76.66%	11.23	0.43
South Africa	99.77%	48.6	26.3	7.65%	0.64	0.65
Average	99.63%	52.52	30.33	0.56	7.80	0.33
Standard deviation	0.01	7.20	8.14	0.22	3.46	0.15
**L1**						
**Population/Area**	**Class I**			**Class II**		
	**Coverage ^a^**	**Average Hit ^b^**	**pc90 ^c^**	**Coverage ^a^**	**Average Hit ^b^**	**pc90 ^c^**
Thailand	99.89%	36.79	20.5	44.94%	5.27	0.18
World	99.93%	38.57	21.86	54.64%	5.99	0.22
East Asia	99.97%	41.67	24.06	62.55%	6.16	0.27
Northeast Asia	99.59%	34.36	16.69	39.26%	4.19	0.16
South Asia	98.79%	30.73	11.99	50.63%	6.38	0.2
Southeast Asia	99.89%	36.3	19.51	38.86%	3.74	0.16
Southwest Asia	97.98%	27.26	9.17	28.24%	2.71	0.14
Europe	100.00%	42.49	27.49	58.32%	6.74	0.24
North America	99.89%	35.37	19.23	58.16%	6.39	0.24
South Africa	99.75%	34.02	17.22	1.79%	0.25	1.43
Average	99.57%	35.76	18.77	43.74%	4.78	0.32
Standard deviation	0.01	4.62	5.40	0.18	2.08	0.39

^a^ projected population coverage; ^b^ average number of epitope hits/HLA combinations recognized by the population; ^c^ minimum number of epitope hits/HLA combinations recognized by 90% of the population.

## Data Availability

The original contributions presented in this study are included in the article. Further inquiries can be directed to the corresponding author.

## Supplementary Materials

The following supporting information can be downloaded at https://www.mdpi.com/article/10.3390/ph19050758/s1; Figure S1: Templeton–Crandall–Sing (TCS) haplotype networks illustrating the global evolutionary relationships and population dynamics of the HPV structural *L1* gene; Figure S2: Amino acid composition profile of the major capsid protein (L1) from all HPV strains; Figure S3: Amino acid composition profile of the early oncoprotein E6 from all HPV strains; Figure S4: Amino acid composition profile of the early oncoprotein E7 from all HPV strains; Figure S5: Templeton–Crandall–Sing (TCS) haplotype networks illustrating the global evolutionary relationships and population dynamics of the HPV structural *L2* gene; Figure S6: Templeton–Crandall–Sing (TCS) haplotype networks illustrating the global evolutionary relationships and population dynamics of the HPV structural *E6* gene; Figure S7: Templeton–Crandall–Sing (TCS) haplotype networks illustrating the global evolutionary relationships and population dynamics of the HPV structural *E7* gene; Figure S8: Amino acid composition profile of the minor capsid protein (L2) from all HPV strains; Figure S9: Templeton–Crandall–Sing (TCS) haplotype networks illustrating the global evolutionary relationships and population dynamics of the HPV structural *E1* gene; Figure S10: Templeton–Crandall–Sing (TCS) haplotype networks illustrating the global evolutionary relationships and population dynamics of the HPV structural *E4* gene; Figure S11: Templeton–Crandall–Sing (TCS) haplotype networks illustrating the global evolutionary relationships and population dynamics of the HPV structural *E2* gene; Figure S12: Templeton–Crandall–Sing (TCS) haplotype networks illustrating the global evolutionary relationships and population dynamics of the HPV structural *E5* gene; Figure S13: Amino acid composition profile of the early protein E1 from all HPV strains; Figure S14: Amino acid composition profile of the early protein E2 from all HPV strains; Figure S15: Amino acid composition profile of the early protein E4 from all HPV strains; Figure S16: Amino acid composition profile of the early protein E5 from all HPV strains; Table S1: Reference HPV from PaVE were available from NCBI database; Table S2: The IMGT/HLA datasets used in this study; Table S3: The biological properties of HPV conserved protein for vaccine construction; Table S4: Prediction of Bepipred Linear Epitope of L1 and L2 protein of HPV type 6, 11, 16, 18, 31, 33, 45, 52, and 58; Table S5: Prediction of discontinuous epitope of L1 and L2 protein of HPV types 6, 11, 16, 18, 31, 33, 45, 52, and 58; Table S6: Predicted MHC Class I T-cell epitopes for the Human Papillomavirus type 6, 11, 16, 18, 31, 33, 45, 52, and 58 L1 major capsid protein; Table S7: Predicted MHC Class I T-cell epitopes for the Human Papillomavirus type 6, 11, 16, 18, 31, 33, 45, 52, and 58 L2 major capsid protein; Table S8: Predicted MHC Class II T-cell epitopes and immunogenicity profiles for the HPV types 6, 11, 16, 18, 31, 33, 45, 52, and 58 L1 major capsid protein; Table S9: Predicted MHC Class II T-cell epitopes and immunogenicity profiles for the HPV types 6, 11, 16, 18, 31, 33, 45, 52, and 58 L2 major capsid protein; Table S10: Summary of consensus high-affinity MHC Class I and Class II predicted epitopes for the HPV types 6, 11, 16, 18, 31, 33, 45, 52, and 58 L1 minor capsid protein; Table S11: Summary of consensus high-affinity MHC Class I and Class II predicted epitopes for the HPV types 6, 11, 16, 18, 31, 33, 45, 52, and 58 L2 minor capsid protein. File S1: Haplotype distribution and sequence frequency of HPV genes and HLA alleles. Each haplotype represents a group of identical genetic sequences, followed by the total sequence count and associated GenBank accession numbers. References [69,70,71,72,73,74,75,76,77,78,79,80,81,82,83,84,85,86,87,88,89,90,91,92,93,94,95,96,97,98,99,100,101,102] are cited in Supplementary Materials.

## Abbreviations

The following abbreviations are used in this manuscript:

HPV	Human papillomavirus
dsDNA	Double-stranded DNA
HR	High-risk
LR	Low-risk
VLPs	Virus-like particles
WGS	Whole-genome sequencing
TCS	Templeton–Crandall–Sing
Leu	Leucine
Ile	Isoleucine
Thr	Threonine
Pro	Proline
HTL	Helper T lymphocyte
HCV	Hepatitis C virus
CTLs	Cytotoxic T-Lymphocytes
Hd	Haplotype diversity
