# Supplementary material for: Evolutionary Dynamics of Human Papillomavirus in Thailand: Immunoinformatic Design of a Synergistic L1/L2 Vaccine Candidate
Source: Pharmaceuticals (Basel). 2026 May 12;19(5):758. doi: 10.3390/ph19050758 (PMC13209937; doi:10.3390/ph19050758)
Supplement: Supplementary file 1 [file pharmaceuticals-19-00758-s001.zip › Supplementary All FigureS.pdf]

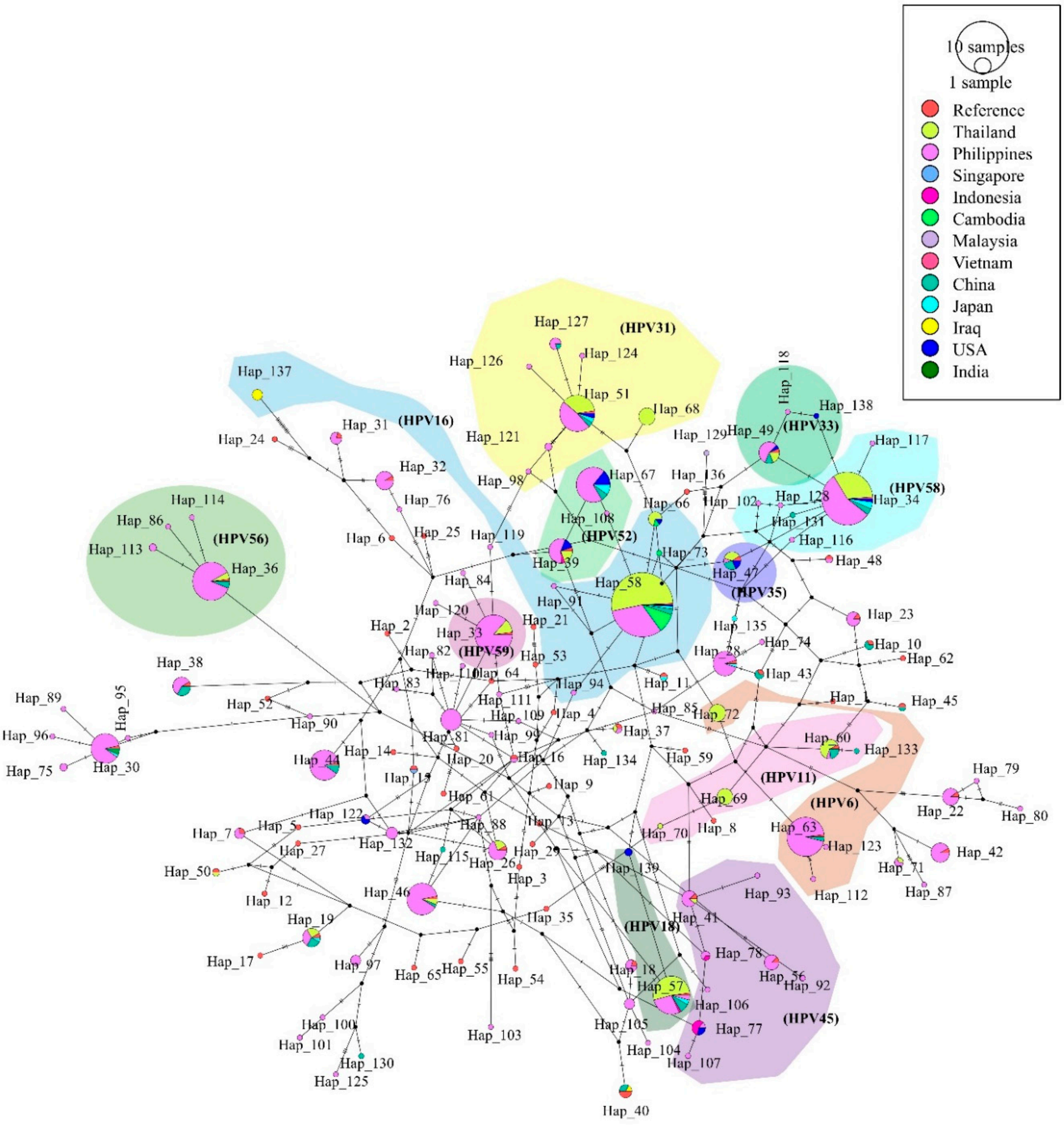

Figure S1. Templeton–Crandall–Sing (TCS) haplotype networks illustrating the global evolutionary relationships and population dynamics of the HPV structural *L1* gene. Each circle (node) represents a distinct genetic haplotype, with the size of the circle proportional to the frequency of that haplotype within the global dataset, connected by genetic mutations.

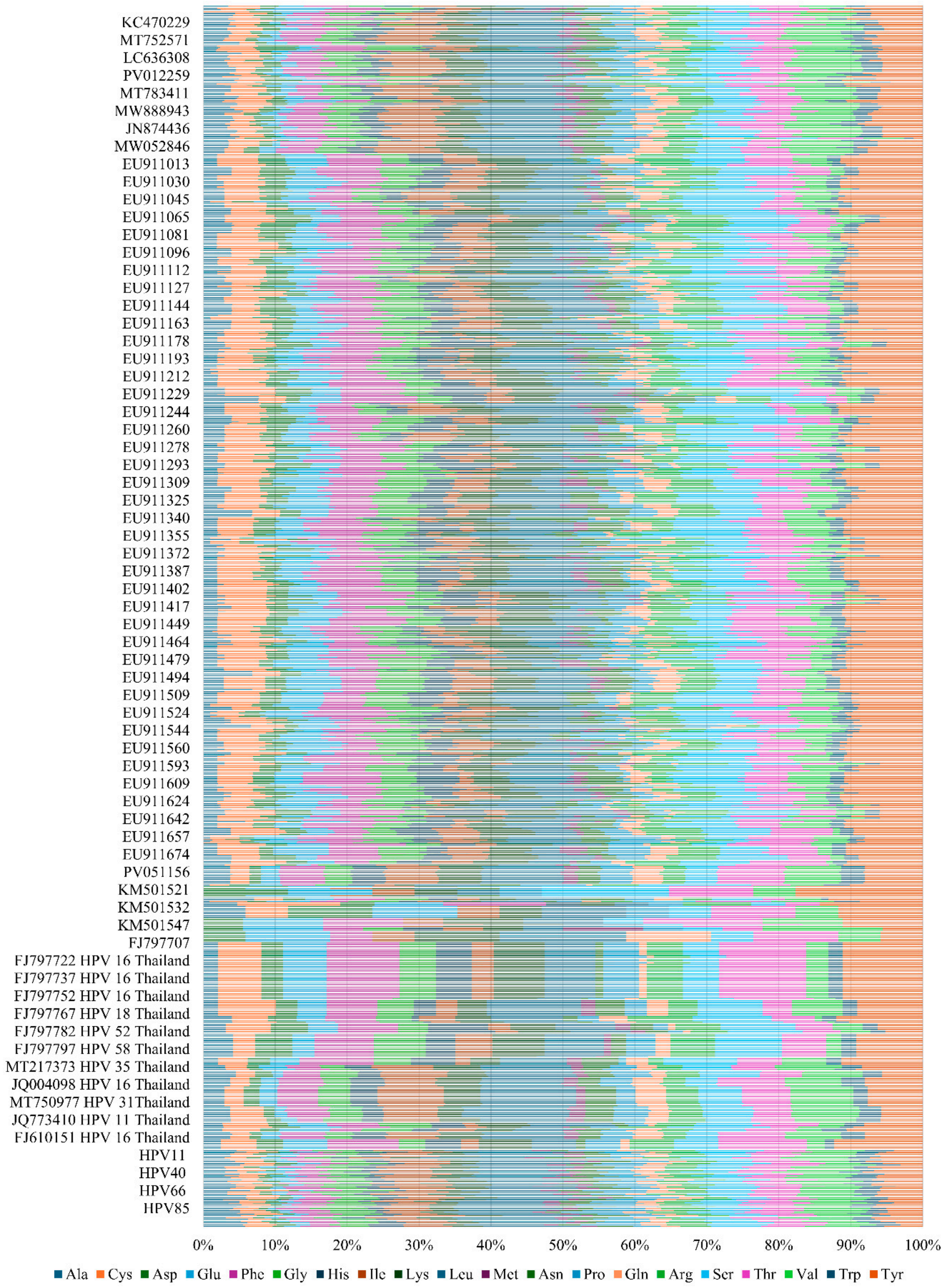

Figure S2. Amino acid composition profile of the major capsid protein (L1) from all HPV strains. The chart illustrates the percentage frequency of each amino acid residue across all L1 protein-coding sequences.

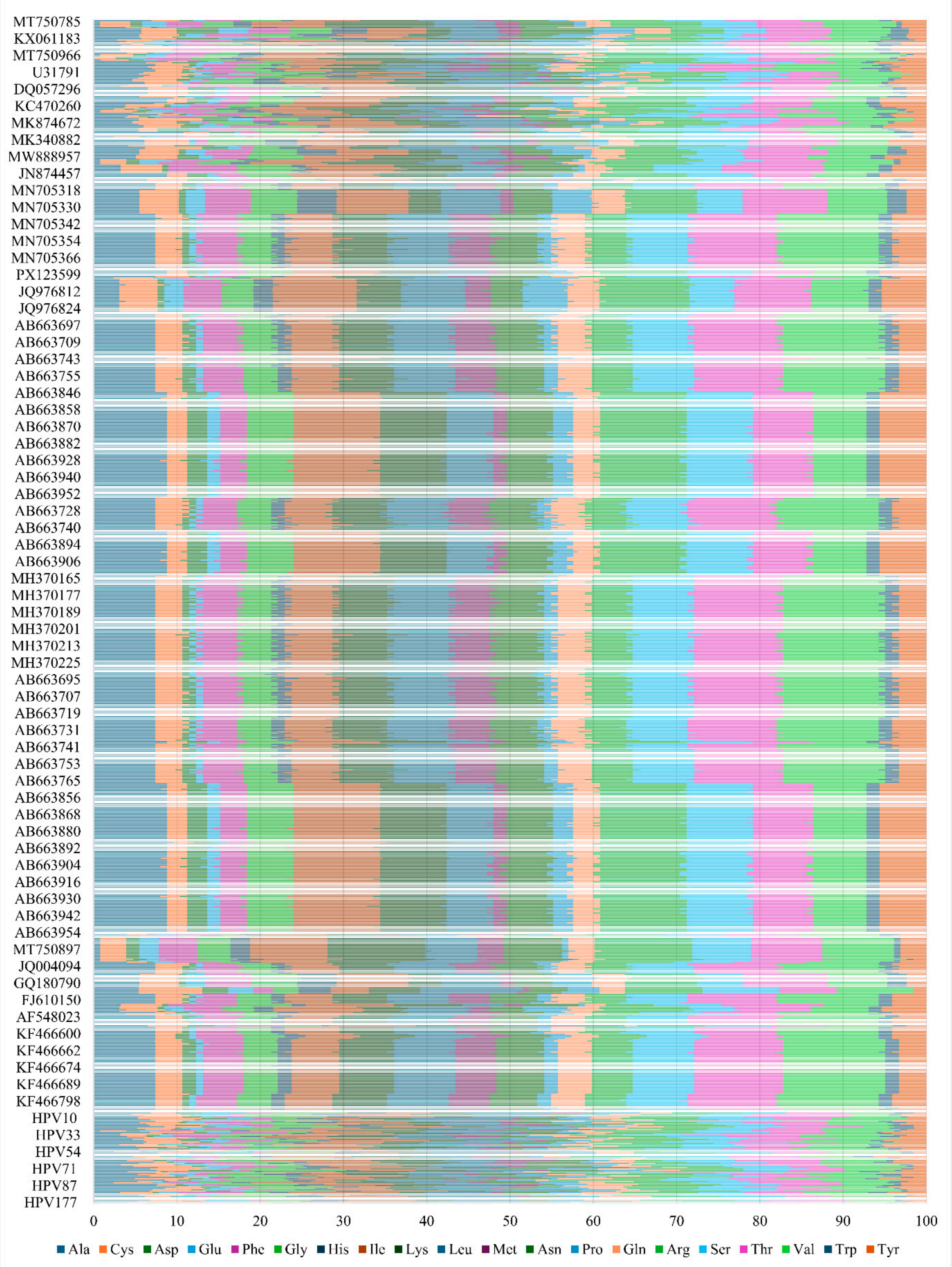

Figure S3. Amino acid composition profile of the early oncoprotein E6 from all HPV strains. The chart illustrates the percentage frequency of each amino acid residue across all E6 protein-coding sequences.

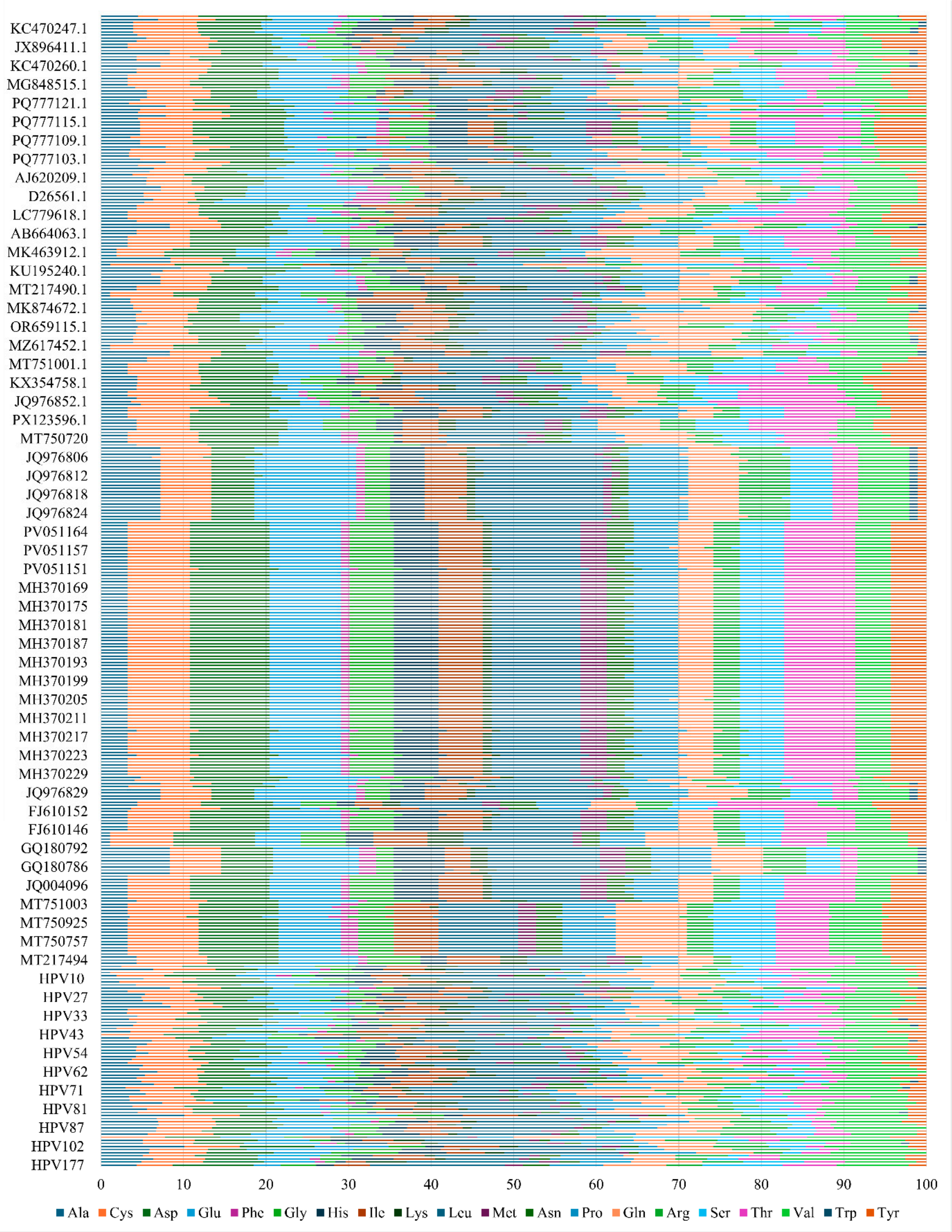

Figure S4. Amino acid composition profile of the early oncoprotein E7 from all HPV strains. The chart illustrates the percentage frequency of each amino acid residue across all E7 protein-coding sequences.

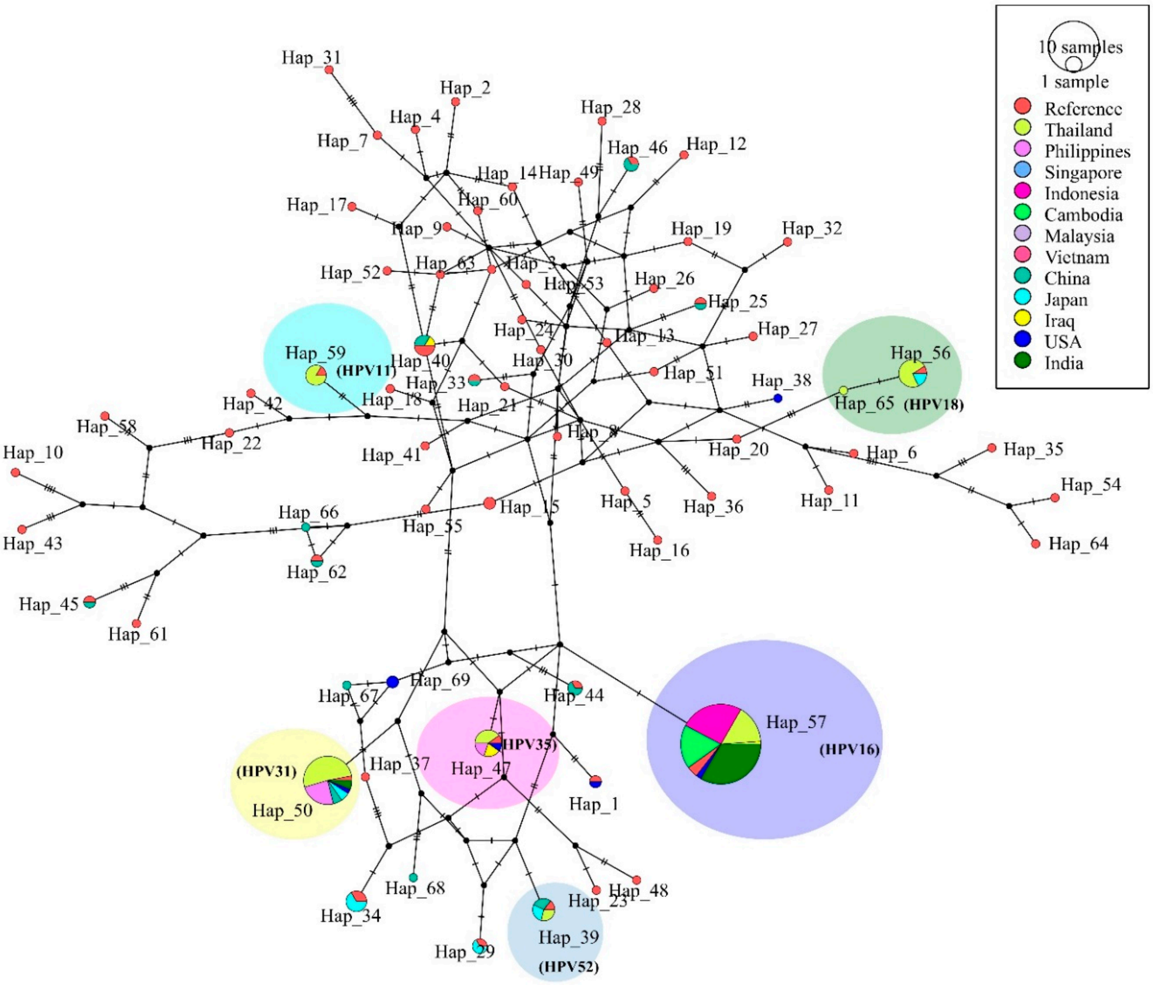

Figure S5. Templeton–Crandall–Sing (TCS) haplotype networks illustrating the global evolutionary relationships and population dynamics of the HPV structural *L2* gene. Each circle (node) represents a distinct genetic haplotype, with the size of the circle proportional to the frequency of that haplotype within the global dataset connected by genetic mutations.

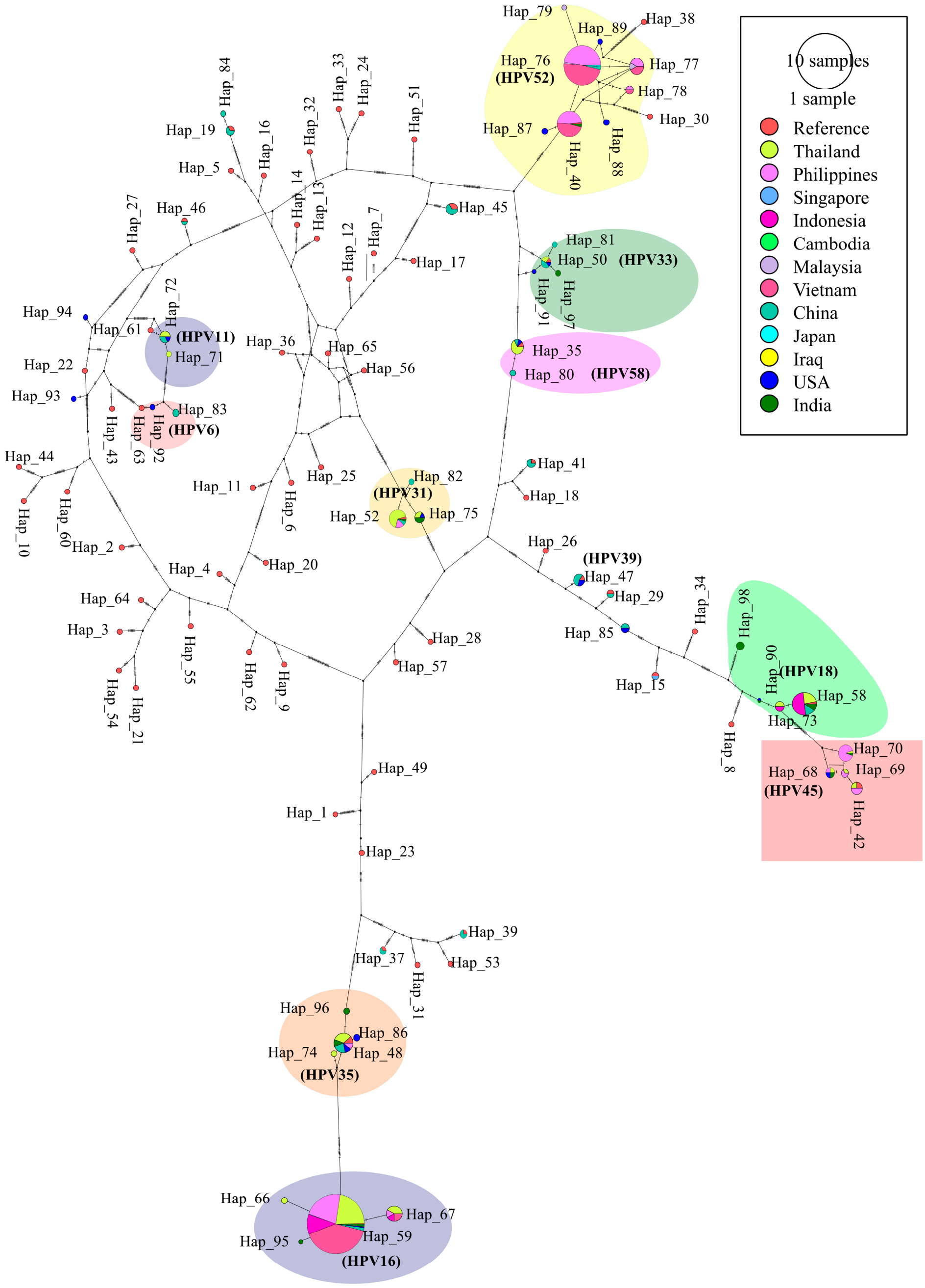

Figure S6. Templeton–Crandall–Sing (TCS) haplotype networks illustrating the global evolutionary relationships and population dynamics of the HPV structural *E6* gene. Each circle (node) represents a distinct genetic haplotype, with the size of the circle proportional to the frequency of that haplotype within the global dataset connected by genetic mutations.

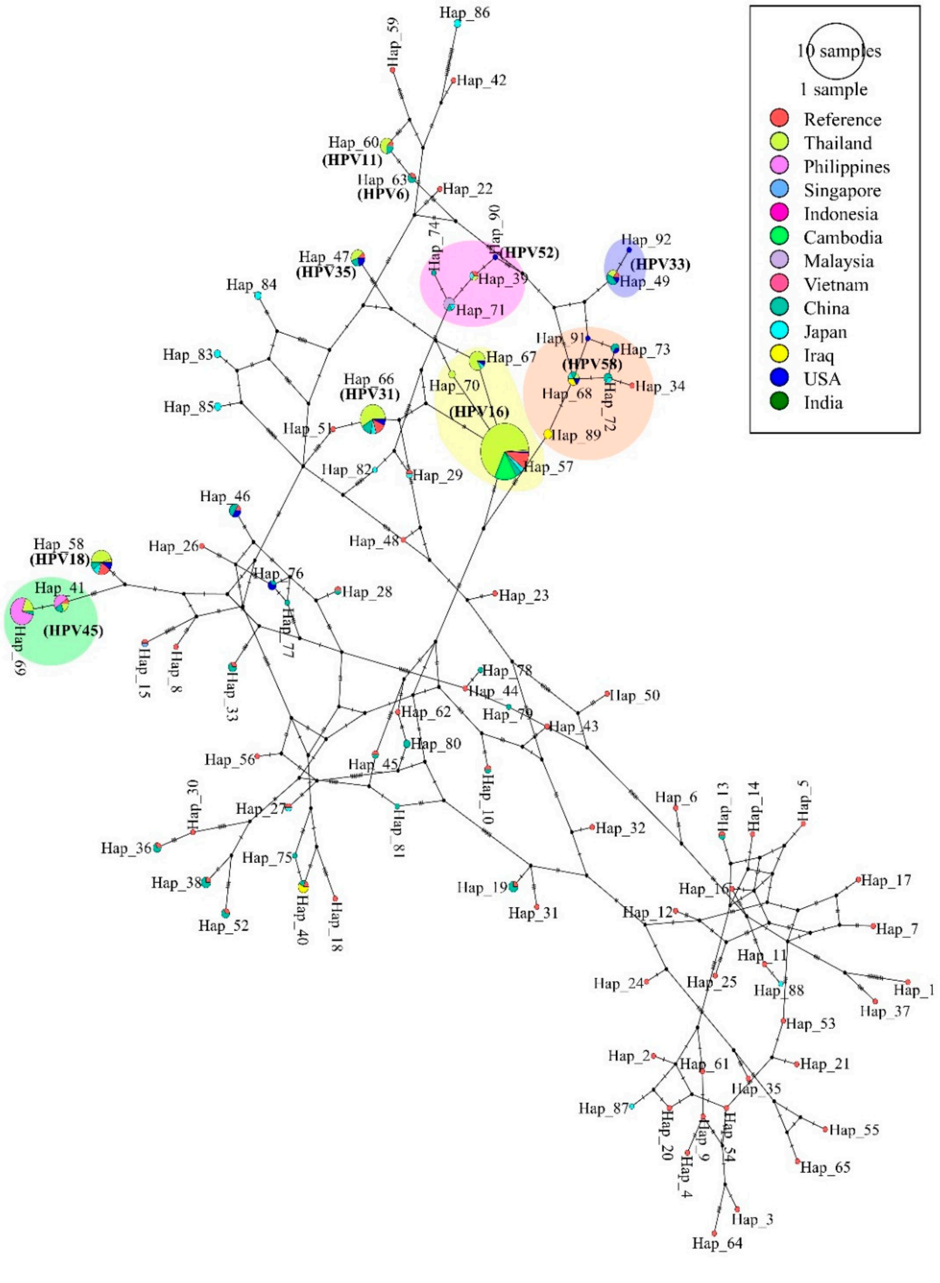

Figure S7. Templeton–Crandall–Sing (TCS) haplotype networks illustrating the global evolutionary relationships and population dynamics of the HPV structural *E7* gene. Each circle (node) represents a distinct genetic haplotype, with the size of the circle proportional to the frequency of that haplotype within the global dataset connected by genetic mutations.

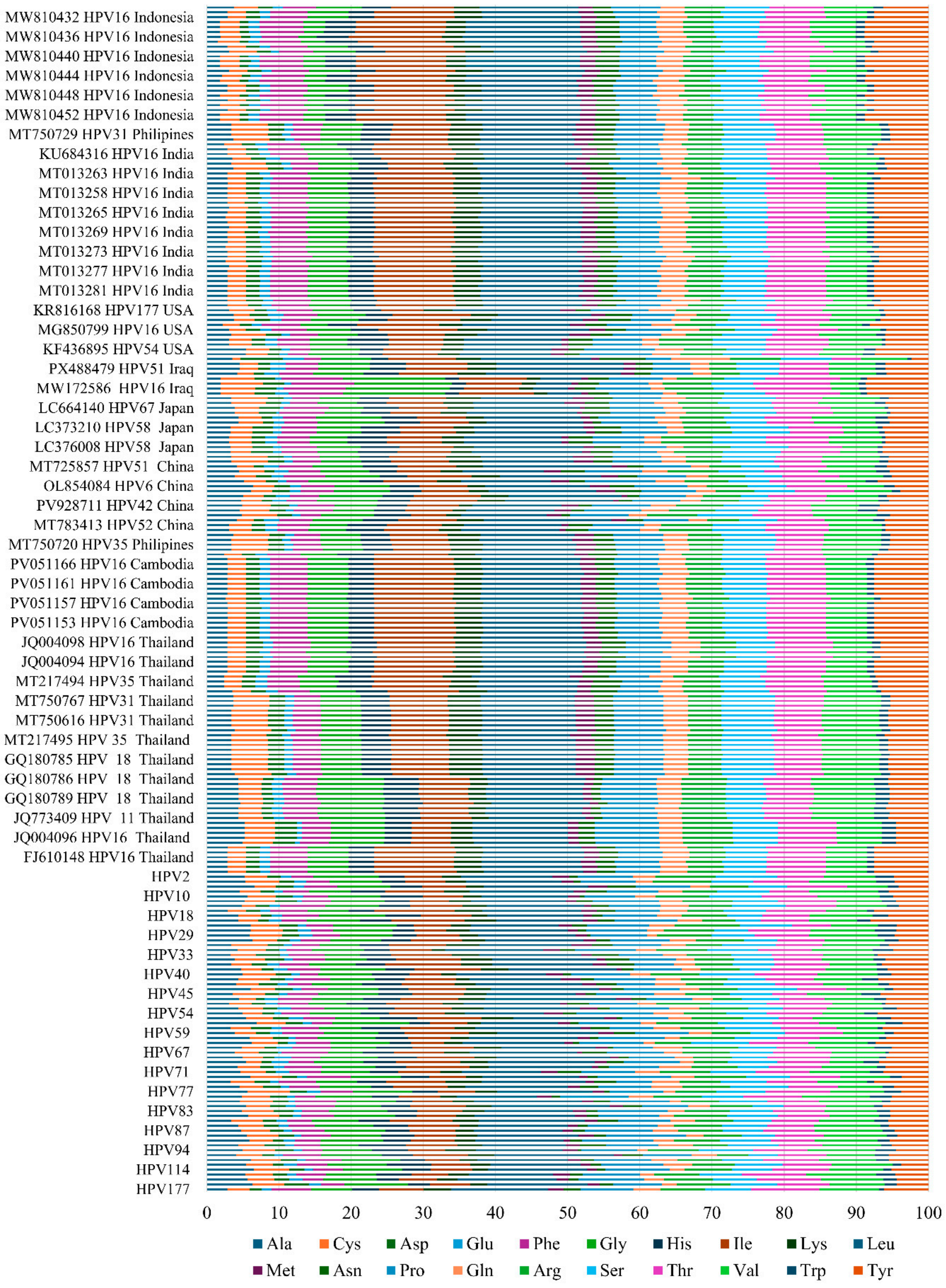

Figure S8. Amino acid composition profile of the minor capsid protein (L2) from all HPV strains. The chart illustrates the percentage frequency of each amino acid residue across all L2 protein-coding sequences.

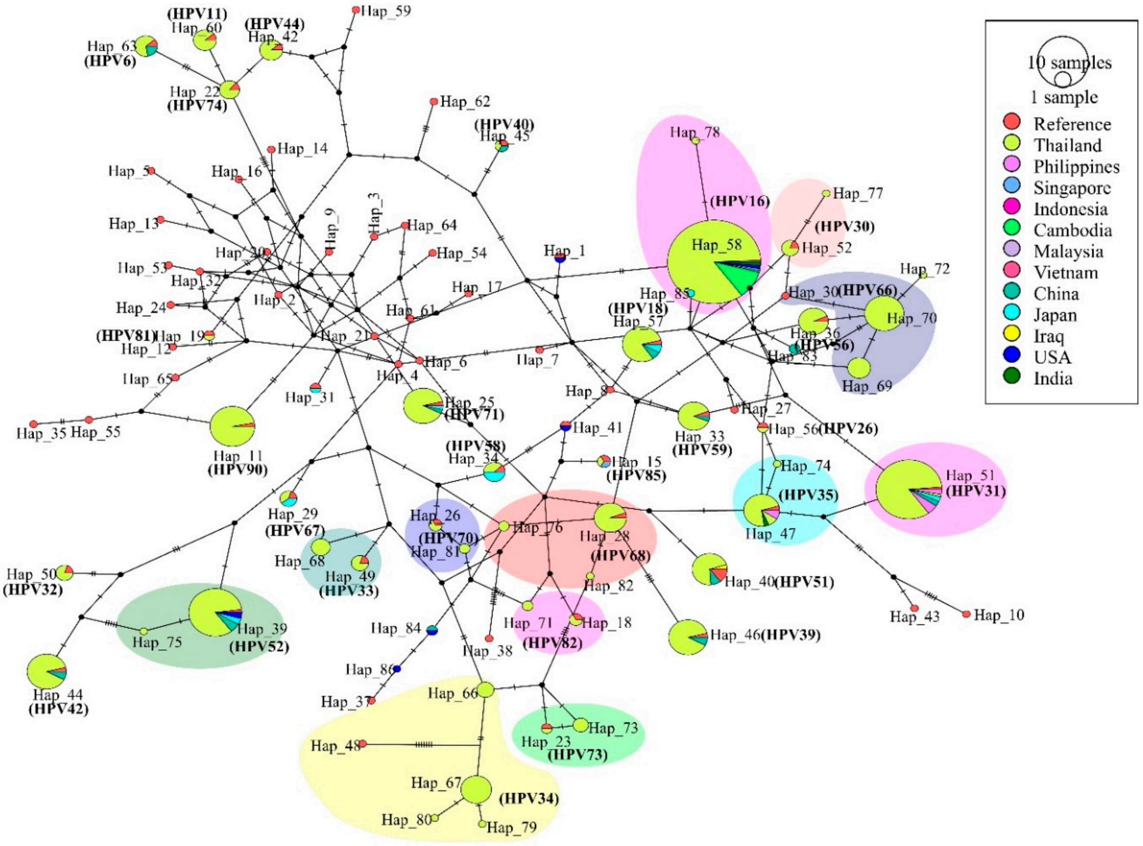

Figure S9. Templeton–Crandall–Sing (TCS) haplotype networks illustrating the global evolutionary relationships and population dynamics of the HPV structural *E1* gene. Each circle (node) represents a distinct genetic haplotype, with the size of the circle proportional to the frequency of that haplotype within the global dataset connected by genetic mutations.

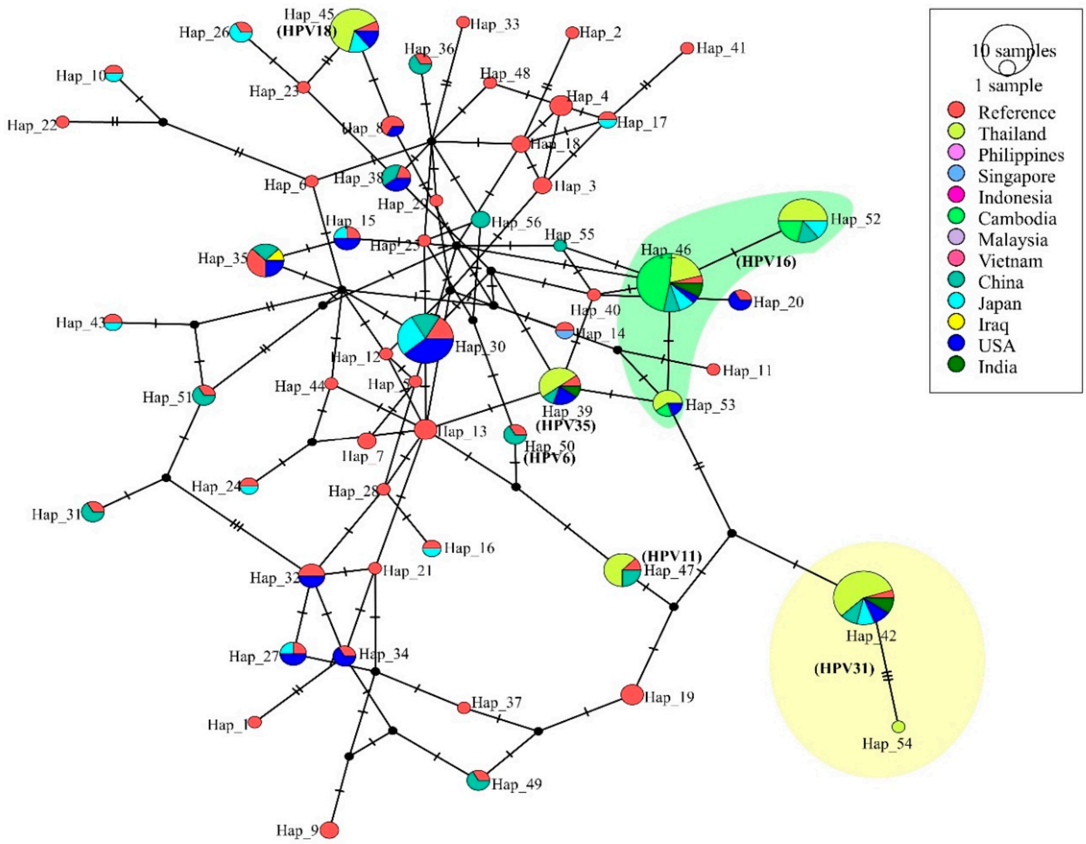

Figure S10. Templeton–Crandall–Sing (TCS) haplotype networks illustrating the global evolutionary relationships and population dynamics of the HPV structural *E4* gene. Each circle (node) represents a distinct genetic haplotype, with the size of the circle proportional to the frequency of that haplotype within the global dataset connected by genetic mutations.

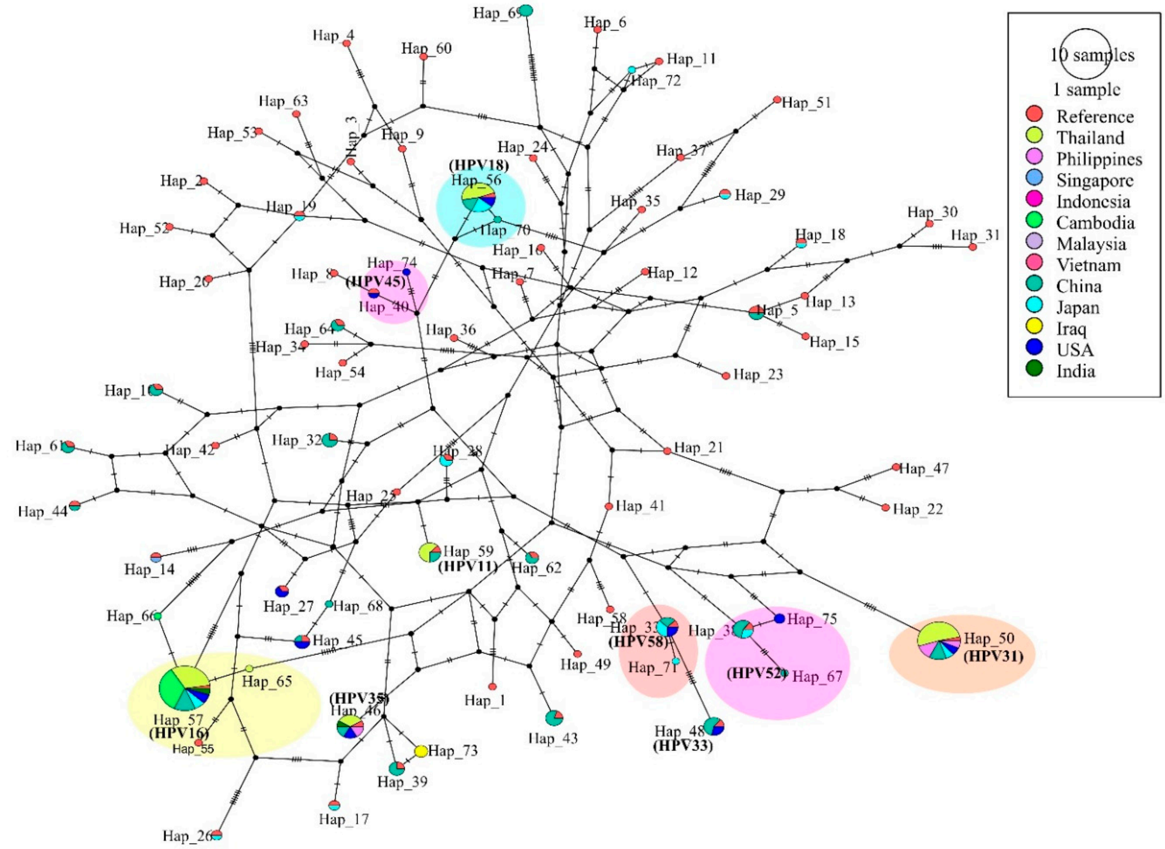

Figure S11. Templeton–Crandall–Sing (TCS) haplotype networks illustrating the global evolutionary relationships and population dynamics of the HPV structural *E2* gene. Each circle (node) represents a distinct genetic haplotype, with the size of the circle proportional to the frequency of that haplotype within the global dataset connected by genetic mutations.

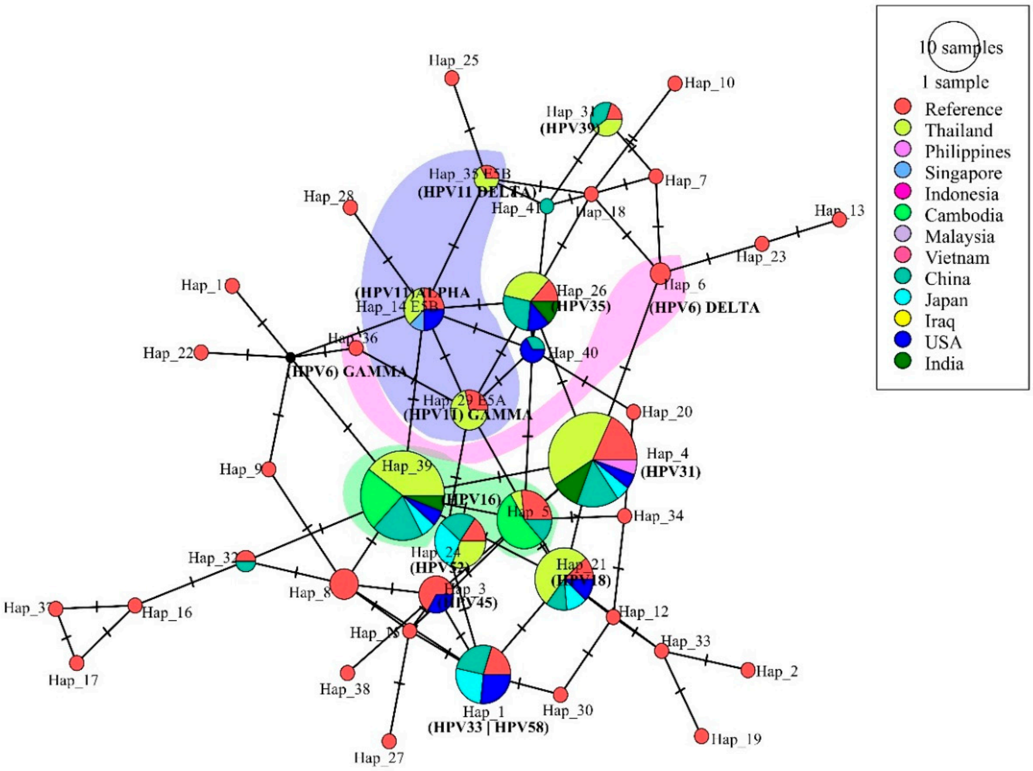

Figure S12. Templeton–Crandall–Sing (TCS) haplotype networks illustrating the global evolutionary relationships and population dynamics of the HPV structural *E5* gene. Each circle (node) represents a distinct genetic haplotype, with the size of the circle proportional to the frequency of that haplotype within the global dataset connected by genetic mutations.

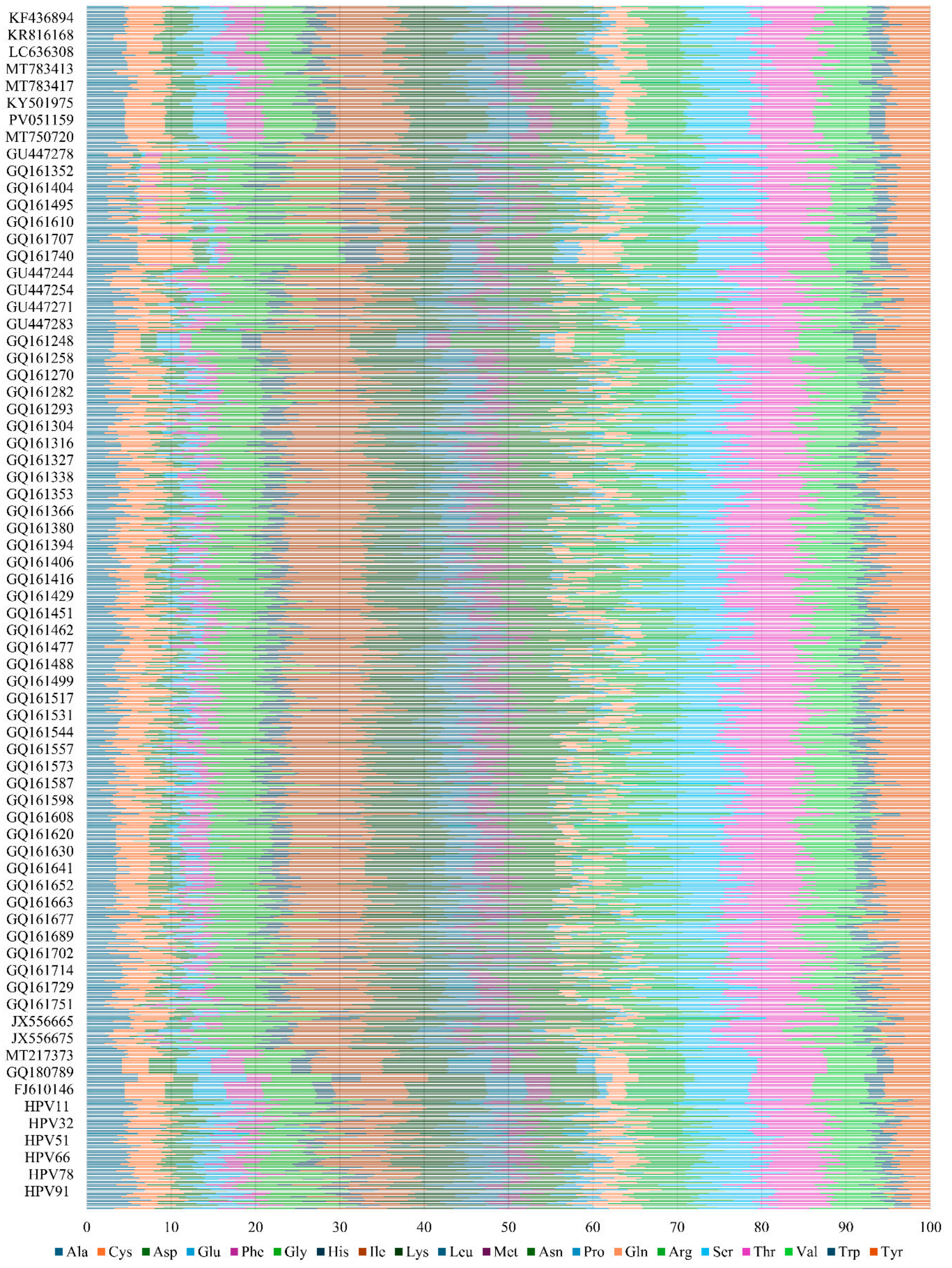

Figure S13. Amino acid composition profile of the early protein E1 from all HPV strains. The chart illustrates the percentage frequency of each amino acid residue across all E1 protein-coding sequences.

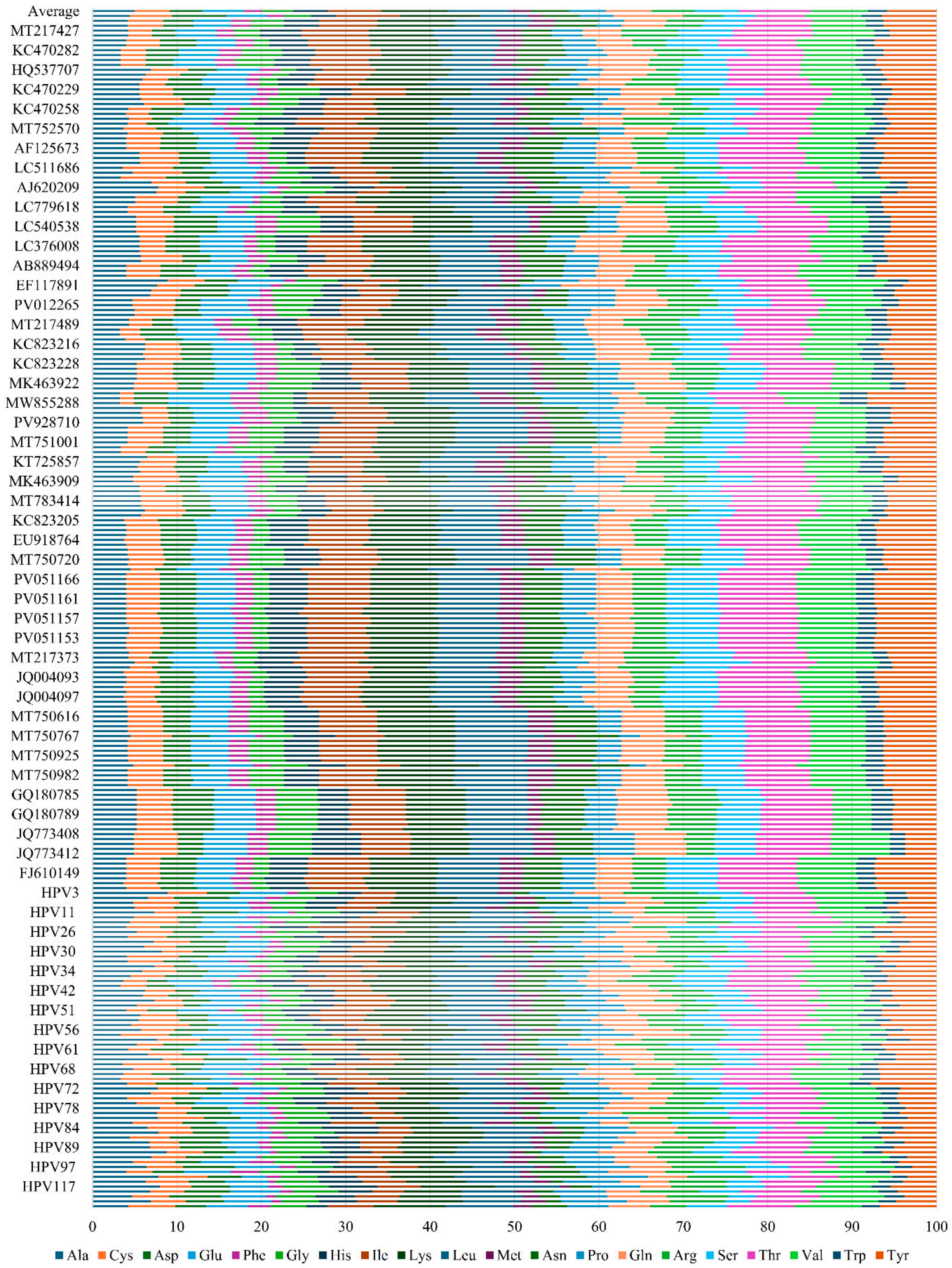

Figure S14. Amino acid composition profile of the early protein E2 from all HPV strains. The chart illustrates the percentage frequency of each amino acid residue across all E2 protein-coding sequences.

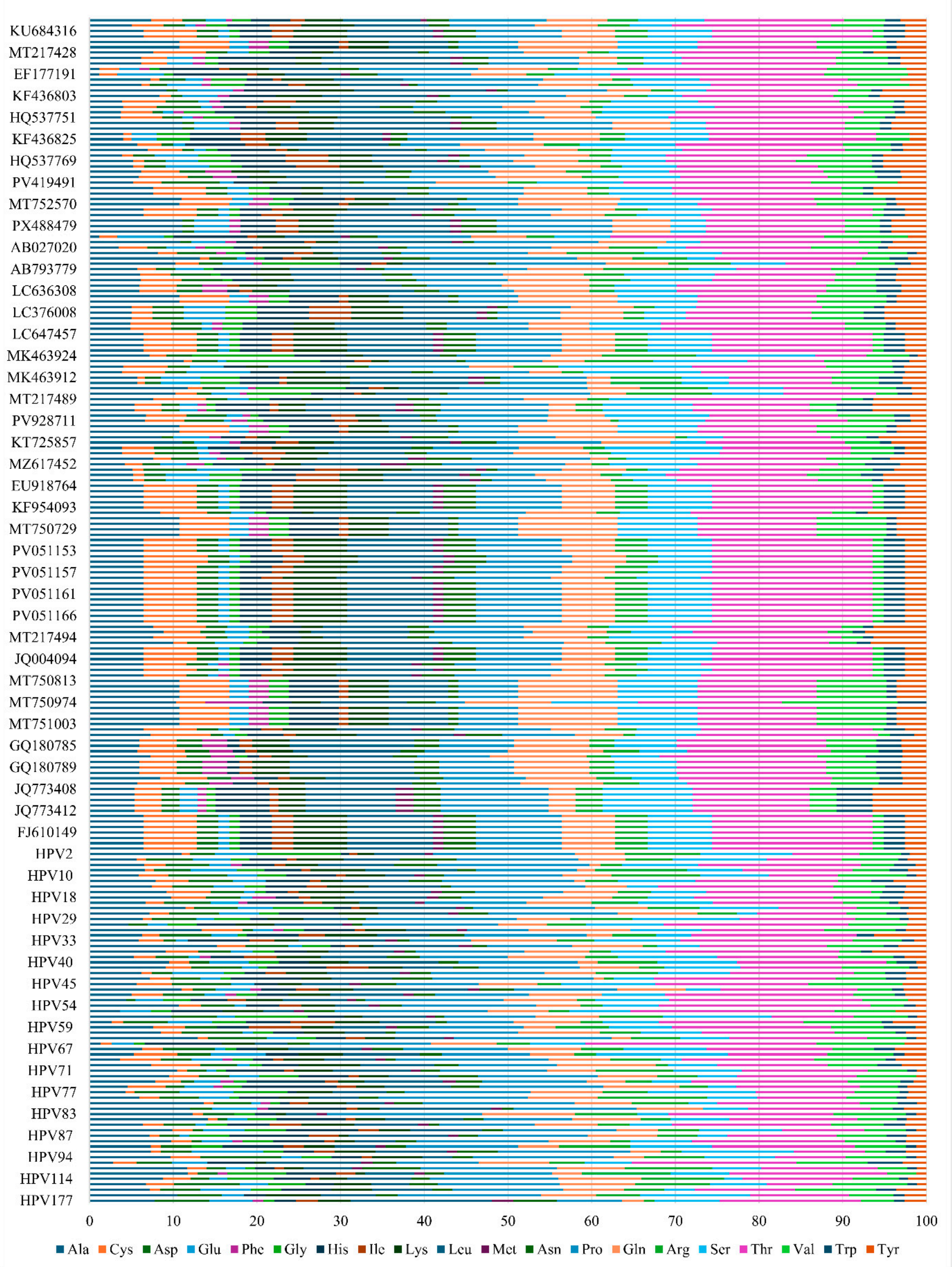

Figure S15. Amino acid composition profile of the early protein E4 from all HPV strains. The chart illustrates the percentage frequency of each amino acid residue across all E4 protein-coding sequences.

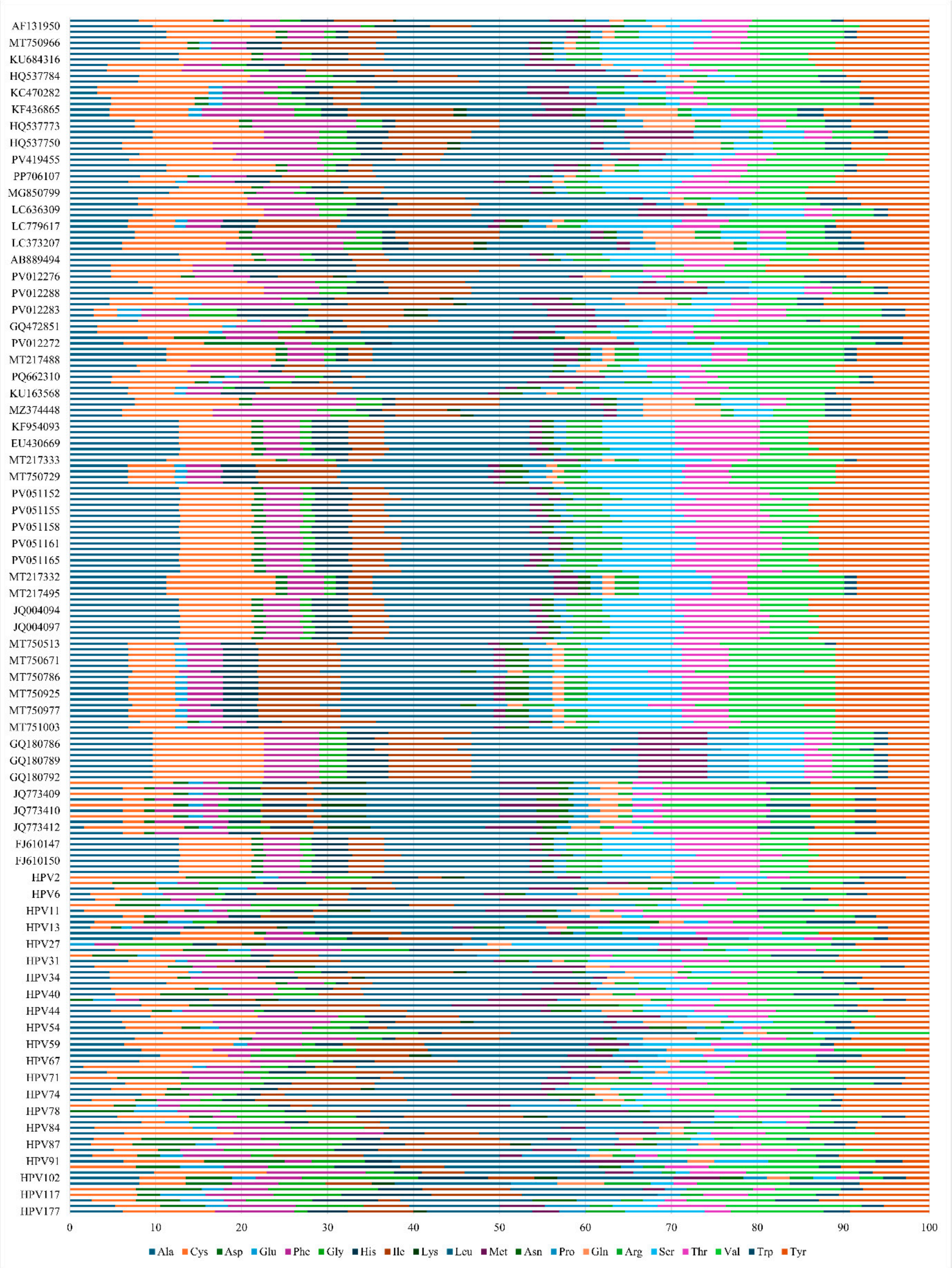

Figure S16. Amino acid composition profile of the early protein E5 from all HPV strains. The chart illustrates the percentage frequency of each amino acid residue across all E5 protein-coding sequences.
